# METTL3-mediated m^6^A modification is required for cerebellar development

**DOI:** 10.1371/journal.pbio.2004880

**Published:** 2018-06-07

**Authors:** Chen-Xin Wang, Guan-Shen Cui, Xiuying Liu, Kai Xu, Meng Wang, Xin-Xin Zhang, Li-Yuan Jiang, Ang Li, Ying Yang, Wei-Yi Lai, Bao-Fa Sun, Gui-Bin Jiang, Hai-Lin Wang, Wei-Min Tong, Wei Li, Xiu-Jie Wang, Yun-Gui Yang, Qi Zhou

**Affiliations:** 1 State Key Laboratory of Stem Cell and Reproductive Biology, Institute of Zoology, Chinese Academy of Sciences, Beijing, China; 2 University of Chinese Academy of Sciences, Beijing, China; 3 Key Laboratory of Genomic and Precision Medicine, Collaborative Innovation Center of Genetics and Development, Beijing Institute of Genomics, Chinese Academy of Sciences, Beijing, China; 4 Sino-Danish College, University of Chinese Academy of Sciences, Beijing, China; 5 Key Laboratory of Genetic Network Biology, Institute of Genetics and Developmental Biology, Chinese Academy of Sciences, Beijing, China; 6 State Key Laboratory of Environmental Chemistry and Ecotoxicology, Research Center for Eco-Environmental Sciences, Chinese Academy of Sciences, Beijing, China; 7 Institute for Stem Cell and Regeneration, Chinese Academy of Sciences, Beijing, China; 8 Department of Pathology, Center for Experimental Animal Research, Institute of Basic Medical Sciences, Chinese Academy of Medical Sciences and Peking Union Medical College, Beijing, China; The University of Chicago, United States of America

## Abstract

*N*^6^-methyladenosine (m^6^A) RNA methylation is the most abundant modification on mRNAs and plays important roles in various biological processes. The formation of m^6^A is catalyzed by a methyltransferase complex including methyltransferase-like 3 (METTL3) as a key factor. However, the in vivo functions of METTL3 and m^6^A modification in mammalian development remain unclear. Here, we show that specific inactivation of *Mettl3* in mouse nervous system causes severe developmental defects in the brain. *Mettl3* conditional knockout (cKO) mice manifest cerebellar hypoplasia caused by drastically enhanced apoptosis of newborn cerebellar granule cells (CGCs) in the external granular layer (EGL). METTL3 depletion–induced loss of m^6^A modification causes extended RNA half-lives and aberrant splicing events, consequently leading to dysregulation of transcriptome-wide gene expression and premature CGC death. Our findings reveal a critical role of METTL3-mediated m^6^A in regulating the development of mammalian cerebellum.

## Author summary

*N*^6^-methyladenosine (m^6^A) is an abundant modification in mRNA molecules and regulates mRNA metabolism and various biological processes, such as cell fate control, early embryonic development, sex determination, and diseases like diabetes and obesity. Adenosine methylation is regulated by a large methyltransferase complex and by demethylases, as well as by other binding proteins. METTL3 is one of the core subunits of the methyltransferase complex catalyzing m^6^A formation. However, the role of METTL3-mediated m^6^A in mammalian brain development remains unclear mainly because of the lack of specific spatiotemporal knockout animal models, as conventional METTL3 knockout in mice leads to early embryonic death. In this study, we specifically inactivated METTL3 in the developing mouse brain. We detected a drastic depletion of m^6^A accompanied by severe developmental defects in the cerebellum of these mice. Further analysis established that METTL3-mediated m^6^A participates in cerebellar development by controlling mRNA stability of genes related to cerebellar development and apoptosis and by regulating alternative splicing of pre-mRNAs of synapse-associated genes.

## Introduction

Multiple layers of epigenetic modifications play essential roles in neuronal development and brain function in mammals through highly coordinated epigenetic regulatory mechanisms [1–5], such as DNA methylation and demethylation [6–8], histone modifications [9, 10], and noncoding RNAs [11–13]. Additionally, RNA modifications have recently been recognized as a new layer of epigenetic regulation, among which *N*^6^-methyladenosine (m^6^A) is the one being most extensively studied. m^6^A formation is catalyzed by a methyltransferase complex including methyltransferase-like 3 (METTL3), methyltransferase-like 14 (METTL14), and Wilms’ tumor 1-associating protein (WTAP), among which METTL3 functions as the catalytic subunit [14, 15]. As the most abundant and reversible modification on mRNAs, m^6^A has been proven to play key roles in regulating RNA stability [16] and RNA splicing [17], as well as mRNA translation efficiency [18–22].

Many essential biological processes are known to be regulated by m^6^A, including cell fate determination [23, 24] and embryonic development [24, 25, 26]. In neuronal systems, m^6^A has been shown to be a dynamic modification and increases in adulthood [27], suggesting a potential function in neural plasticity and brain function. Previous studies have shown that conventional knockout of *Mettl3* in mice leads to early embryonic lethality [24], and specific depletion of m^6^A in the nervous system by conditional knockout of *Mettl14* results in extended cell cycle and altered neurogenesis in embryonic mouse cortex, through regulating the decay of neurogenesis-related transcripts [28]. Furthermore, loss of m^6^A eraser, the fat mass and obesity-associated protein (FTO), leads to impaired adult neurogenesis and cognition ability [29]. FTO is enriched in axons, and specifically silencing axonal FTO inhibits axon elongation by suppressing growth associated protein 43 (*GAP*-43) [30]. Nerve lesion induces the elevation of m^6^A level, and loss of METTL14 or m^6^A binding protein YTH N6-methyladenosine RNA binding protein 1 (YTHDF1) inhibits axonal regeneration [31]. All these studies indicate the important roles of m^6^A signaling in neuronal development and neurogenesis.

The cerebellum offers an ideal model to study neurogenesis, as it contains a limited number of neuronal categories, and different types of cerebellar neurons are generated in a timely order, precisely scheduled by progenitors. The developing cerebellum consists of the external granular layer (EGL), molecular layer (ML), Purkinje cell layer (PCL), and internal granular layer (IGL) [32, 33]. It has been shown that cerebellar granule cell progenitors (GCPs) proliferate in EGL, then migrate through ML to IGL to differentiate into granule cells, generating parallel fibers and forming synaptic connections with Purkinje cells [33]. The EGL, as the exclusive source for cerebellar granule cells (CGCs) [32, 34], gradually becomes thinner and disappears around 21 days postpartum (P21) in mice [33]. Although many studies have indicated the function of epigenetic modifications in cerebellar development, little is known about the roles of m^6^A signaling in the development of the cerebellum and its associated behavioral phenotype. In the present work, we specifically inactivated *Mettl3* in the developing mouse brain using the *Nestin-Cre* mediated *Mettl3* conditional knockout model. Depletion of METTL3 in the brain caused severe developmental defects in both the cortical and cerebellar regions. Further analysis demonstrated drastic apoptosis of newborn CGCs, which is partially responsible for the severe cerebellar hypoplasia and a series of ataxia-like movement disorders. Furthermore, loss of m^6^A by inactivation of *Mettl3* led to extended half-lives of mRNAs from cerebellar development- and apoptosis-associated genes, as well as aberrant mRNA splicing events on synapse-associated genes, which consequently induced inappropriate cell differentiation and cell death, indicating that METTL3-mediated m^6^A serves as a key regulator in modulating the development of the central nervous system at the posttranscriptional level in mammals.

## Results

### Characterization of *Mettl3* conditional knockout mice

Consistent with a previous report [35], we detected ubiquitous occupation of METTL3 in various brain regions by immunostaining (S1A Fig), indicating that METTL3 might be involved in the development of mammalian brains. To investigate the functions of METTL3-mediated m^6^A in brain development, we introduced two *loxP* sites into the *Mettl3* genomic region flanking exons 2–4, a region encoding the methyltransferase catalytic domain in B6D2F1 mice by CRISPR/Cas9 system-assisted homologous recombination (S1B Fig). The resulting *Mettl3*^*flox/flox*^ mice were crossed with *Nestin-Cre* transgenic mice to generate *Mettl3* conditional knockout mice (genotype *Mettl3*^*flox/flox*^;*Nestin-Cre*, named as cKO) (S1B and S1C Fig). Genotyping results confirmed the specific deletion of *Mettl3* exons 2–4 in the brain tissue of newborn cKO mice, whereas the *Mettl3* coding sequence in their tail tissue remains intact (S1C–S1G Fig). Immunohistochemical staining (S1A Fig) and western blot (S1H Fig) further confirmed that the cKO mice failed to produce METTL3 protein and its isoforms in the whole brain, whereas in other nonneural tissues, such as liver, METTL3 protein remained unperturbed. Littermates of the cKO mice with the *Mettl3*^*flox/flox*^ or *Mettl3*^*flox/+*^ genotypes were used as the controls (Ctrl).

**Fig 1:**
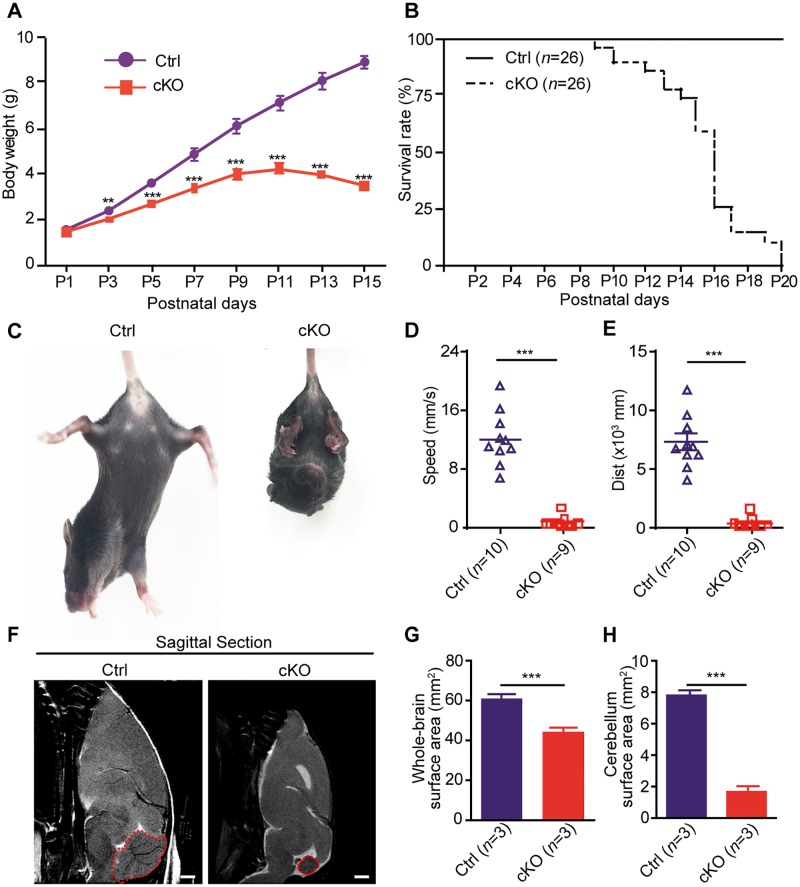
Characterization of the *Mettl3* conditional knockout mice. (A) Body weight changes of the Ctrl and cKO mice during the first 2 wk after birth. (B) Kaplan–Meier survival curves showing the survival rates of both Ctrl and cKO mice. (C) Tail suspension test of both Ctrl and cKO mice. (D, E) Open field test of Ctrl and cKO mice for their speed (D) and total traveled distance (E). (F) MRI of mice sagittal brain section. Scale bar, 1 mm. (G, H) Surface areas of the whole brain (G) and cerebellum (H) of Ctrl and cKO mice. Sagittal section. The data underlying this figure can be found in S1 Data. Data shown are means ± SEM, *n* = 4. **p-value < 0.01, ***p-value < 0.001, Student *t* test. cKO, *Mettl3* conditional knockout; Ctrl, control; *Mettl3*, methyltransferase-like 3; MRI, magnetic resonance imaging; SEM, standard error of the mean.

The cKO pups had a significantly decreased body weight growth rate as compared to that of the Ctrl (Fig 1A) and were only approximately half the size of their Ctrl littermates by P14 (S2A Fig). The majority of cKO pups died before P20 without artificial feeding (Fig 1B). In addition, the cKO mice demonstrated balance disorders and altered gaits after P7 that became severe at around P14 (S1 Movie). When lifted by the tail, the cKO mice displayed dramatic tremors with tightly curled legs and forward bended head (Fig 1C and S2 and S3 Movies), demonstrating typical features of cerebellar ataxia [36]. We then analyzed the behavior of the mice in an open field test; the cKO mice only generated intermittent and slow movements with much reduced speed and moving distance, and were seldom capable of moving out of the central region (Fig 1D and 1E and S2B–S2D Fig). The smaller body size, premature death, and ataxia-like movement disorders indicated severe developmental defects in the cKO mouse brain. We then performed magnetic resonance imaging (MRI) analysis and identified smaller brain sizes and enlarged ventricles in the brains of *Mettl3* cKO mice (S2E Fig), which is consistent with the results in another m^6^A depletion model via *Nestin-Cre* mediated conditional knockout of *Mettl14* [28]. Furthermore, in addition to the cortex, we also observed shrunk and unstructured cerebellums in the *Mettl3* cKO mice (Fig 1F–1H). It is tempting to speculate that the developmental defects in the cerebellums caused by depletion of METTL3 might contribute to the movement disorders of the cKO pups. Ultra high-pressure liquid chromatography tandem-mass spectrometry (UHPLC-MS/MS) analysis showed that the m^6^A modification on mRNAs of cKO mouse brain tissues were nearly wiped out (S2F Fig), which indicated that the brain developmental defects might be caused by m^6^A depletion.

### *Mettl3* conditional knockout causes cerebellar hypoplasia in mice

**Fig 2:**
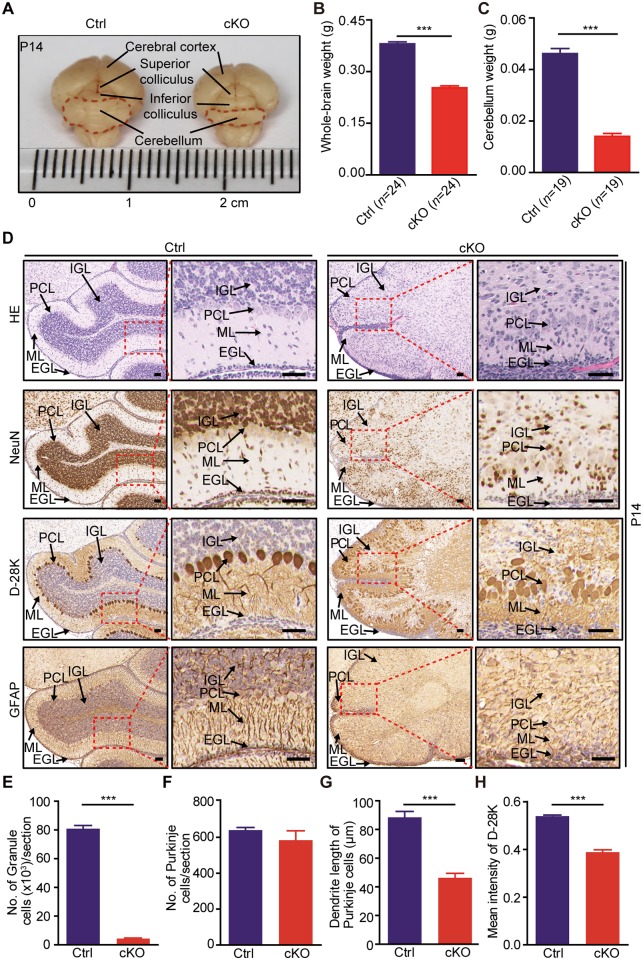
*Mettl3* conditional knockout causes cerebellar hypoplasia in mice. (A) Macromorphological comparison between brains of the Ctrl and cKO mice. (B, C) Weight of the whole brain (B) and cerebellum (C) of the Ctrl and cKO mice. (D) Histological abnormalities of cerebellum in cKO mice shown by HE staining, NeuN, D-28K, and GFAP immunohistochemical staining. Scale bar, 200 μm. (E, F) Density difference of granule cells (E) and Purkinje cells (F) in the Ctrl and cKO mouse cerebellums. (G, H) Dendrite length (G) and mean calbindin D-28K staining intensity. (H) Difference of Purkinje cells in the Ctrl and cKO cerebellums. P14 mice were used in all studies. Data related to this figure are shown in S1 Data. Data shown are means ± SEM. For cell number count in (E) and (F), *n* = 3; for dendrite length and D-28K staining intensity in (G) and (H), *n* = 10. ***p-value < 0.001, Student *t* test. cKO, *Mettl3* conditional knockout; Ctrl, control; D-28K, calbindin 1; EGL, external granular layer; GFAP, glial fibrillary acid protein; HE, hematoxylin and eosin; IGL, internal granular layer; *Mettl3*, methyltransferase-like 3; ML, molecular layer; NeuN, neuronal nuclei; PCL, Purkinje cell layer; SEM, standard error of the mean.

A previous report has demonstrated that the m^6^A RNA methylation level in mouse cerebellum is generally higher than that in other brain regions [35]. Consistently, our western blot showed relatively higher expressions of METTL3 and METTL14 in the cerebellum than those in the cerebral cortex of wild-type mouse (S3A Fig). UHPLC-MS/MS analysis also detected twice the amount of m^6^A on mRNA from the cerebellum in comparison to that from other brain regions of the wild-type mouse (S3B Fig). We then focused on the effect of METTL3 depletion on the development of the cerebellum. We first systematically investigated the expression pattern of METTL3 in developing mouse cerebellum by immunohistochemical staining, and we found evident and ubiquitous immunostaining signals in the EGL, PCL, ML, and IGL of the cerebellum from embryonic day 16.5 (E16.5) to P14 (S4A–S4C Fig). Interestingly, the METTL3 expression level in the proliferating GCPs within the outer EGL was shown to be relatively lower than that of the newborn CGCs within the inner EGL at P7 (S4B Fig). We confirmed that METTL3 was depleted in the cKO cerebellums as early as E16.5, when no substantial structural defect was seen in the cerebellar region of the cKO mouse brains (S4A–S4C Fig). Macroscopic histological examination showed shrunken size, loss of weight, and remarkable failure in foliation of the cKO cerebellums (Fig 2A–2C). Further immunohistological analysis revealed drastic loss of CGCs in the IGL layer of the cKO cerebellums (Fig 2D and 2E and S4D Fig). In spite of the fact that the number of Purkinje cells in the cKO cerebellums remained almost the same as that in the Ctrl mice, they failed to maintain the well-organized single-cell layer (Fig 2D and 2F and S4D Fig). In addition, Purkinje cells in cKO cerebellums had much shorter dendrites and exhibited reduced calbindin 1 (CABL1, also known as D-28K) staining signal (Fig 2D, 2G and 2H and S4D Fig), indicating defective Purkinje cell maturation in the cKO cerebellums. Glial fibrillary acid protein (GFAP) staining also showed that the Bergmann glia lost their scaffold organization pattern in the cKO cerebellums (Fig 2D and S4D Fig). Based on the above, we concluded that depletion of METTL3 caused severe cerebellar hypoplasia in mice, indicating that m^6^A played important roles in regulating not only the development of the cortex [28] but also the cerebellar region of the brain.

### Depletion of METTL3 induces apoptosis of newborn granule cells

**Fig 3:**
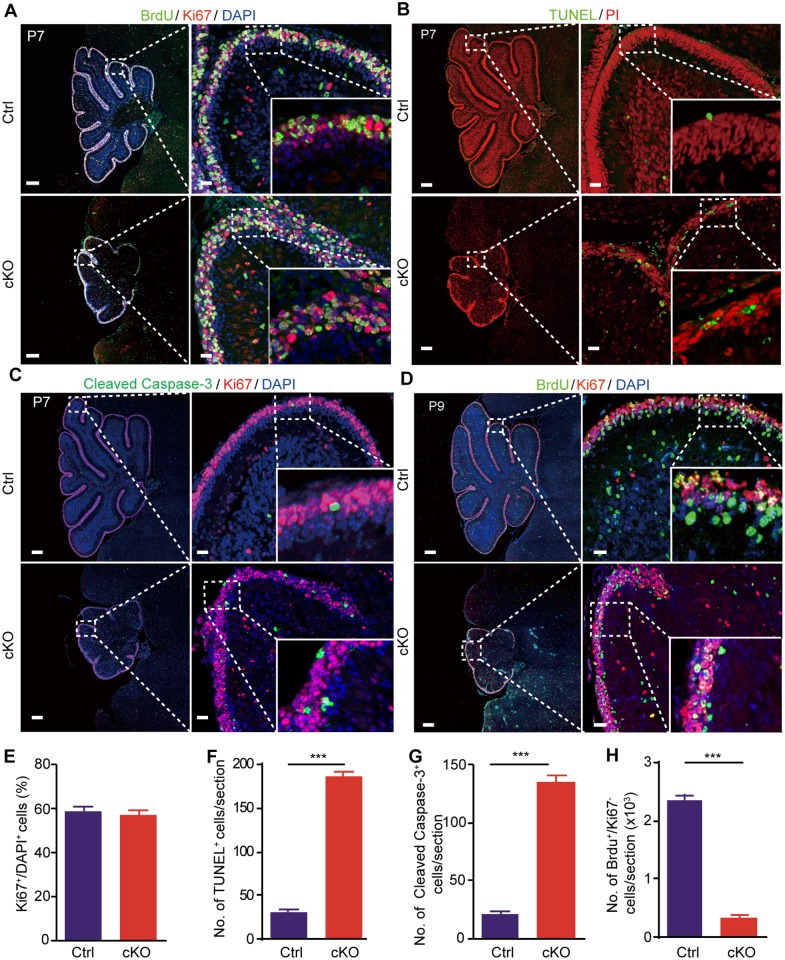
*Mettl3* conditional knockout induces apoptosis of newborn granule cells. (A) BrdU (green), Ki67 (red), and DAPI (blue) immunofluorescent staining of Ctrl and cKO mouse cerebellums 2 h after BrdU injection. (B) TUNEL (green) and PI (red) staining of P7 Ctrl and cKO mouse cerebellums. (C) Cleaved Caspase-3 (green), Ki67 (red), and DAPI (blue) immunofluorescent staining of P7 Ctrl and cKO mouse cerebellums. (D) BrdU (green), Ki67 (red), and DAPI (blue) immunofluorescent staining of Ctrl and cKO mouse cerebellums 48 h after BrdU injection. (E) Proportion of Ki67^+^/DAPI^+^ cells in the EGL of P7 Ctrl and cKO mice. (F, G) Density of TUNEL^+^ (F) and Cleaved Caspase-3^+^ (G) cells in P7 Ctrl and cKO mouse cerebellums. (H) Density of BrdU^+^/Ki67^−^ cells in the EGL of Ctrl and cKO mice 48 h post–BrdU injection. Scale bars in (A–D), 200 μm for left panels; 25 μm for right panels. Data related to this figure are shown in S1 Data. Data shown are means ± SEM. Three sections of each sample were analyzed. ***p-value < 0.001, Student *t* test. BrdU, bromodeoxyuridine; cKO, *Mettl3* conditional knockout; Ctrl, control; EGL, external granular layer; Ki67, antigen identified by monoclonal antibody Ki 67; *Mettl3*, methyltransferase-like 3; PI, Propidium iodide; SEM, standard error of the mean.

We further assessed the proliferation potential of GCPs using an in vivo bromodeoxyuridine (BrdU) labeling assay. The cerebellar tissues were dissected from P7 mice 2 h post–BrdU injection and costained with BrdU/antigen identified by monoclonal antibody Ki 67 (Ki67)/DAPI. The numbers of Ki67^+^ cells exhibited no difference between the Ctrl and cKO samples, indicating that GCPs maintain the ability to proliferate in cKO EGLs (Fig 3A and 3E), although with a slightly reduced rate (S5A and S5B Fig). However, both TUNEL and Cleaved Caspase-3 immunostaining revealed a significantly increased cell apoptosis rate in cKO cerebellum, especially in the EGL region (Fig 3B, 3C, 3F and 3G). Histological examination also detected a large number of karyorrhexis and karyopyknosis, the characteristic nuclear forms of apoptotic cells, in the EGL of cKO cerebellums (S5C Fig). Consistent with the enhanced apoptotic signals, the BrdU^+^/Ki67^−^ cells, representing postmitotic newborn granule neurons, were drastically reduced in cKO cerebellums at 48 h post–BrdU injection (Fig 3D and 3H). These data demonstrated that the severe depletion of CGCs in cKO cerebellums mainly resulted from the abnormal postmitotic apoptosis of newborn granule cells.

### m^6^A depletion induces dramatic gene expression changes in the cerebellum

**Fig 4:**
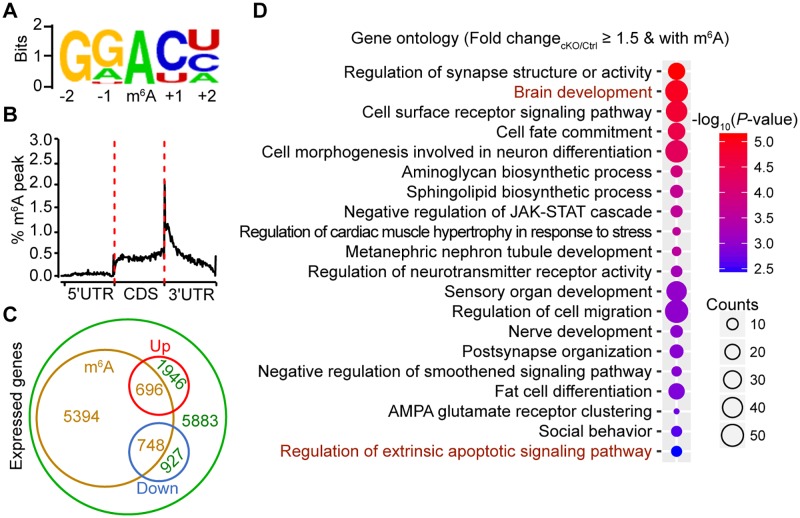
m^6^A depletion induces dramatic gene expression changes in the cerebellum. (A) The most enriched sequence motif of m^6^A peaks in mRNAs from P7 mouse cerebellums. (B) Distribution of m^6^A peaks along the 5′UTR, CDS, and 3′UTR regions of total cerebellar mRNAs from P7 Ctrl mice after normalized with length. (C) Venn diagram representing the relationships between cerebellar expressed genes and m^6^A modification. Green circle represents all expressed genes in P7 Ctrl mouse cerebellums, brown circle represents genes with m^6^A modifications in P7 Ctrl mouse cerebellums, red circle represents genes with up-regulated expression in P7 *Mettl3* cKO mouse cerebellums as compared to the Ctrl, and blue circle represents genes with down-regulated expression in P7 *Mettl3* cKO mouse cerebellums as compared to the Ctrl. Numbers represent the counts of genes in each group. (D) Significantly enriched (*p*-value ≤ 0.01, Benjamini–Hochberg multiple testing correction) GO terms of genes with up-regulated expression in P7 cKO cerebellums and with m^6^A peaks. CDS, coding sequence; cKO, *Mettl3* conditional knockout; GO, gene ontology; JAK-STAT, Janus kinase (JAK), Signal Transducer and Activator of Transcription protein (STAT); m^6^A, *N*^6^-methyladenosine.

To further investigate the underlying mechanisms for the cerebellar developmental defects in *Mettl3* cKO mice, we performed RNA sequencing (RNA-seq) and m^6^A sequencing (m^6^A-seq) using mRNAs extracted from the cerebellums of Ctrl and cKO mice at both P7 and P14. Over 10,000 and 15,000 differential m^6^A peaks were identified in the cerebellar mRNAs of P7 and P14 Ctrl mice, respectively, whereas in *Mettl3* cKO mice, only a few hundred (background level) differential m^6^A peaks were detected (S1, S2 and S3 Tables). Consistent with previous reports [16, 37], the m^6^A peaks in cerebellar RNAs were also enriched in the regions with RRACH motif (Fig 4A and S6A Fig) and tended to occur near stop codons and within 3′UTRs of mRNAs (Fig 4B and S6B Fig). Among the 15,614 expressed genes in the cerebellum of the P7 Ctrl mice, transcripts of 6,838 genes contain m^6^A peaks (Fig 4C). Because the presence of m^6^A peaks could facilitate mRNA degradation [16], we focused on the 696 genes with both m^6^A peak loss and elevated expression in the cKO cerebellums (Fig 4C), as this group of transcripts was likely stabilized after m^6^A depletion. Gene ontology analysis revealed that most of these genes were enriched in neural development-associated biological processes and the apoptotic signaling pathway (Fig 4D and S7A and S7B Fig). Similar results were also obtained from the m^6^A-seq and RNA-seq data at P14 (S6A–S6D and S7C and S7D Figs).

### m^6^A depletion enhances the stability of development- and apoptosis-associated gene transcripts

**Fig 5:**
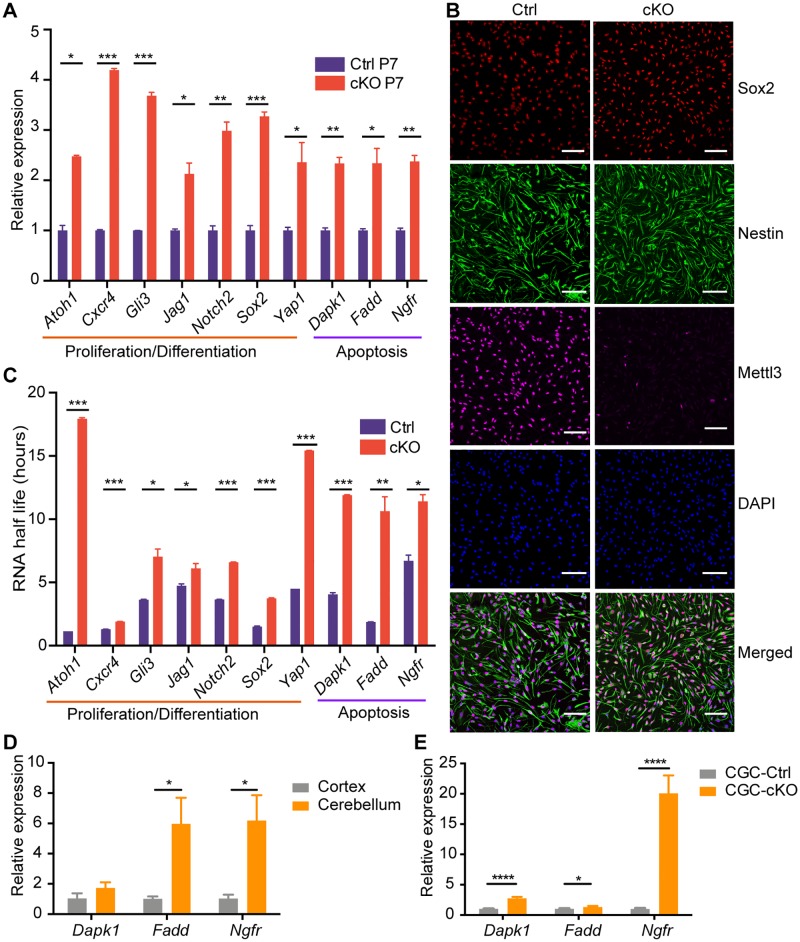
m^6^A depletion enhances the stability of development- and apoptosis-associated gene transcripts. (A) qRT-PCR results confirmed the up-regulated expression in P7 cKO cerebellums of selected genes. The major functions of detected genes are shown under the lines. (B) Immunostaining for Sox2 (red), Nestin (green), and Mettl3 (purple) in neural stem cell lines established from Ctrl and *Mettl3* cKO neonatal mice. Scale bar, 100 μm. (C) The RNA half-lives of genes detected in (A). Values and error bars in (A) and (C) represent the mean and SEM of three independent experiments. (D) qRT-PCR results confirmed higher expression levels of apoptosis-promoting genes (*Dapk1*, *Fadd*, *Ngfr*) in cerebellums of P7 wild-type mice than those in the cortex. (E) qRT-PCR results confirmed the up-regulated expression of the apoptosis-promoting genes in the cKO CGCs. Data related to this figure are shown in S1 Data. Data shown are means ± SEM. *p-value < 0.05, **p-value < 0.01, ***p-value < 0.001, ****p-value < 0.0001, Student *t* test. CGC, cerebellar granule cell; cKO, *Mettl3* conditional knockout; Ctrl, control; Mettl3, methyltransferase-like 3; qRT-PCR, quantitative reverse transcription-polymerase chain reaction; RT-PCR, reverse transcription-polymerase chain reaction; SEM, standard error of the mean.

Quantitative reverse transcription-polymerase chain reaction (qRT-PCR) analysis confirmed the up-regulation of genes with m^6^A loss (S8A and S8C Fig) in cKO cerebellums, including essential factors for cerebellar development such as *Atoh1*, *Cxcr4*, *Notch2* and its ligand *Jag1* [32, 38–41], as well as neuronal apoptosis such as *Dapk1*, *Fadd*, and *Ngfr* [42–44] (Fig 5A and S6E Fig). We further measured the degradation rates of these mRNAs in neural stem cell (NSC) lines established from the Ctrl and cKO neonatal mice (Fig 5B) using the actinomycin-D mediated transcription inhibition assay. We detected prolonged mRNA half-lives of these development- and apoptosis-associated genes in the cKO NSCs (Fig 5C). Because there were no reported apoptosis phenotypes in the developing cortex [28], we compared the expression levels of the apoptosis-promoting genes that showed extended half-lives with the depletion of m^6^A in both the cerebellar region and the cortex of wild-type mice by quantitative polymerase chain reaction (qPCR), and the results displayed higher expression levels of these genes in the cerebellar region than in the cortex (Fig 5D). Furthermore, in vitro cultured CGCs isolated from P7 mice showed elevated expression levels of these genes with the depletion of METTL3 (Fig 5E). All lines of evidence indicated that these apoptosis-promoting genes functioned predominantly in the cerebellum CGCs rather than in the cortex. Our data demonstrated that the depletion of m^6^A resulted in a dysregulated gene expression profile during the development of the cerebellum, especially for genes related to cerebellar development and cell apoptosis.

### m^6^A depletion induces exon exclusion in synapse-associated genes

**Fig 6:**
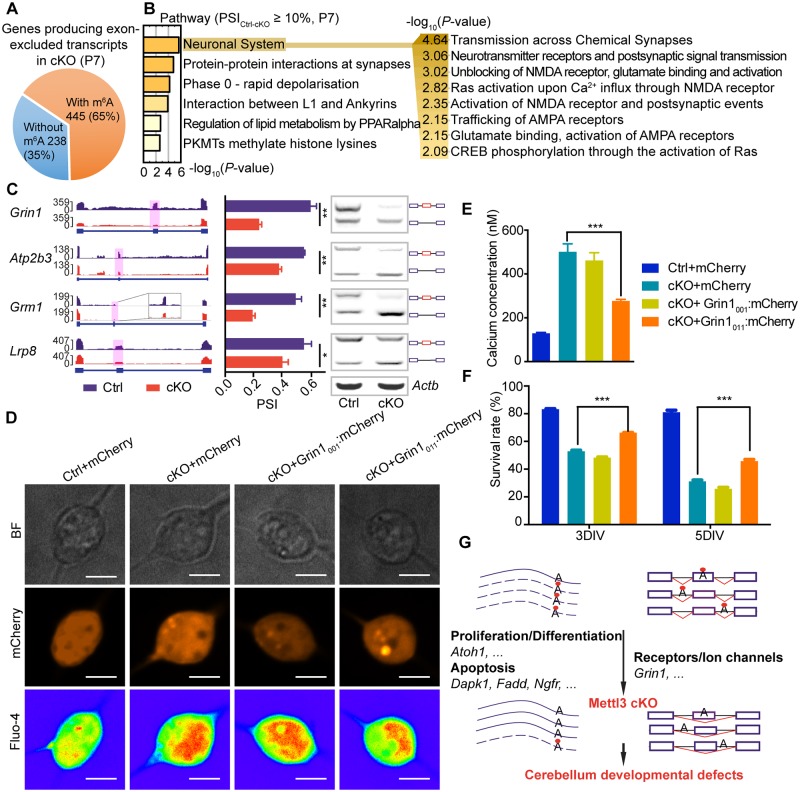
m^6^A depletion induces exon exclusion in synapse-associated genes. (A) Genes producing exon-excluded transcripts in P7 cKO cerebellums as compared with the Ctrl. (B) Enriched pathways of genes producing exon-excluded transcripts in P7 cKO cerebellums as compared with the Ctrl. Enriched pathways under the “Neuronal system” are expanded on the right. (C) Production of exon-excluded transcripts of major synapse-associated genes in P7 cKO cerebellums. Left, IGV tracks displaying the RNA-seq reads coverage in P7 Ctrl (blue) and cKO (red) cerebellum, the excluded exons are shaded with pink columns. Middle, calculated exon-exclusion percentage in P7 cKO cerebellums by the PSI value. Right, semiquantitative PCR detection of exon-excluded transcripts in P7 cKO cerebellums. (D) Images of the CGCs in the calcium detection assay. *Grin1*_*001*_ or *Grin1*_*011*_ was introduced into the cKO CGCs by electroporation and was co-expressed with *mCherry* (red). *Fluo-4* (rainbow) indicated the cellular calcium concentration in the CGCs. Scale bar, 5 μm. (E) Statistical analysis of the cellular calcium concentration in the calcium detection assay. (F) CCK8 assay shows the survival rate of the Ctrl CGCs with overexpression of *mCherry* and cKO CGCs with overexpression of *Grin1*_*001*_ or *Grin1*_*011*_ in 3 DIV and 5 DIV. (G) Schematic diagram illustrating the mechanisms underlying the regulatory functions of METTL3-mediated m^6^A modification in cerebellar development. Red dots represent m^6^A modification. The box and line symbols in the right part of the diagram represent pre-spliced RNAs, with exons shown as boxes and introns shown as black lines. Exons in post-spliced RNAs are connected by red lines. Further information about this figure can be found in S1 Data. The data were represented as means ± SEM. For calcium concentration detection, *n* = 10; for CCK8 assay, *n* = 3. *p-value < 0.05, **p-value < 0.01, ***p-value < 0.001, Student *t* test. AMPA, α-amino-3-hydroxy-5-methyl-4-isoxazolepropionic acid; BF, bright field; CCK8, Cell Counting Kit-8; CGC, cerebellar granule cell; cKO, *Mettl3* conditional knockout; CREB, cAMP responsive element binding protein; Ctrl, control; DIV, days in vitro; *Grin1*_*001*_, C1 region excluded variant of *Grin1*; *Grin1*_*011*_, C1 region included variant of *Grin1*; IGV, integrative genomics viewer; METTL3, methyltransferase-like 3; m^6^A, *N*^*6*^-methyladenosine; NMDA, N-methyl-D-aspartic acid; PKMT, Lysine methyltransferase; PPAR, peroxisome proliferator-activated receptor; PSI, percent spliced in index; RNA-seq, RNA sequencing; SEM, standard error of the mean.

Consistent with a previous report that m^6^A depletion tends to produce exon-excluded transcripts [17], we also found that the majority of exon-excluded transcripts in cKO cerebellums (as compared with the Ctrl) were derived from m^6^A-containing transcripts in the Ctrl at both P7 and P14 (Fig 6A and S9A Fig). Interestingly, genes with exon-excluded transcripts in cKO were enriched in synapse-associated pathways, including transmission across chemical synapses and protein–protein interactions at synapses (Fig 6B and S9B Fig). More specifically, we found that genes associated with the two major glutamate receptors, the N-methyl-D-aspartic acid (NMDA) receptors and the α-amino-3-hydroxy-5-methyl-4-isoxazolepropionic acid (AMPA) receptors, were highly enriched in the pathway analysis (Fig 6B). NMDA receptors were reported to be essential for the maturation and survival of CGCs [44, 45]. We confirmed that the m^6^A-modified exons (S8B and S8C Fig) in these synapse-associated genes, such as *Grin1*, *Atp2b3*, *Grm1*, and *Lrp8*, were excluded in cKO cerebellums by RT-PCR (Fig 6C and S9C Fig). The MiniGene assay confirmed the exclusion of exon 21 (C1 region) of *Grin1* (S10A Fig) and exon 19 of *Lrp8* (S10B Fig) with the depletion of m^6^A by either *Mettl3* knockdown or mutation of the m^6^A modification sites (S10C and S10D Fig), indicating the regulatory role of m^6^A modification in RNA splicing and function of the ion channel associated genes.

The NMDA receptor is a heterodimer of glutamate ionotropic receptor NMDA type subunit 1 (GRIN1) and glutamate ionotropic receptor NMDA type subunit 2 (GRIN2) and is permeable to Ca^2+^ when activated by glutamate. The *Grin1* gene contains three alternatively spliced exons (ASEs), namely N1, C1, and C2, and generates at least eight splicing variant isoforms [46]. The C1 region has been shown to be a regulatory domain for calcium influx and is reported to be affinitive to calmodulin (CaM) [47] and inhibit the NMDA receptor in a Ca^2+^-dependent way [48]. To further investigate the effect of the altered splicing of *Grin1* induced by depletion of m^6^A on the survival of CGCs, we isolated and cultured CGCs from both Ctrl and cKO mouse cerebellums. We overexpressed *Grin1*_*001*_ (C1 excluded variant) or *Grin1*_*011*_ (C1 included variant) in the cKO CGCs by electroporation. We found that cKO CGCs had a much higher intracellular calcium concentration than that of the Ctrl ones, whereas overexpression of *Grin1*_*011*_, but not mCherry or *Grin1*_*001*_, reduced the cellular calcium concentration in the cKO CGCs (Fig 6D and 6E). Consistently, Cell Counting Kit-8 (CCK8) assay detected an increased survival rate of the cKO CGCs with *Grin1*_*011*_ overexpression (Fig 6F). Our findings established that the altered *Grin1* splicing induced by m^6^A depletion resulted in a substantial increase in intracellular calcium concentration and consequently the apoptosis of the CGCs.

## Discussion

It is well established that epigenetic machineries play essential roles in controlling the timing and linage commitment of NSCs during neurogenesis [49, 50]. Epigenetic mechanisms such as DNA methylation and histone modifications regulate brain development and plasticity at the transcriptional level [4, 51]. Additionally, noncoding RNAs and RNA modifications also appeared to add complexities at the epitranscriptomic level in regulating neuronal development and brain function [26, 52–57], and multiple layers of epigenetic modification regulations have been shown to be required for neurogenesis in the developing cerebellum [58–60]. DNA demethylation is highly active during early cerebellum development, and inhibition of DNA demethylation suppresses the circuit formation of developing granular cells [60]. GCPs and glial cells express high levels of histone deacetylase 1 (HDAC1), indicating the potential role of histone modification in neurogenesis of the cerebellum [61]. The microRNAs (miRNAs) have been reported to regulate components of Hedgehog-Patched signaling in normal or transformed GCPs [62]. In addition, alternative transcription combined with alternative splicing gives rise to transcriptomic diversity during cerebellar development [59].

Recent study has reported that specific depletion of m^6^A in the mouse brain by conditional knockout of *Mettl14* causes postponed neurogenesis in the cortex [28]. In our study, we found remarkable cerebellar developmental defects in *Mettl3* conditional knockout mouse model, indicating the indispensable roles of METTL14- and METTL3-mediated m^6^A signaling in neuronal development. In addition, we recognized a severe cerebellar hypoplasia phenotype in *Mettl3* cKO mice. Depletion of m^6^A caused apoptosis of the newborn granule cells, which then resulted in altered Purkinje cell and Bergmann glia architecture. Our findings demonstrated, for the first time, the regulatory function of m^6^A modification in cerebellar development.

The development of the mouse cerebellum is quite distinct from that of the cerebral region of the brain. Neurogenesis dominantly occurs in the embryonic stage in the cortex, while in the cerebellar region, this process mainly occurs in the postnatal 2 wk [32]. A whole transcriptome-wide m^6^A methylation analysis depicted a spatial-specific methylation profile of mouse brain and revealed that m^6^A methylation had a higher level in the cerebellum than that in the cerebral cortex [35], indicating pivotal roles of m^6^A in this brain region. The development of the mouse cerebellum is a highly organized process involving a cooperated set of genetic and epigenetic regulations. By high throughput RNA-seq and m^6^A-seq, we revealed that key developmental genes like *Atoh1* and *Cxcr4* were abnormally up-regulated because of the extended mRNA half-lives induced by m^6^A depletion in the cKO mice.

During the development of CGCs, apoptosis occurs occasionally as a normal process [63], whereas in the cKO mice, in addition to developmental associated genes, we also detected up-regulation of apoptosis-associated genes like *Dapk1* and *Fadd*, with extended half-lives of their mRNAs. Hence the m^6^A modification on mRNAs may play an indispensable role in balancing cell survival and apoptosis by regulating mRNA stability of development- and apoptosis-associated genes.

Comparison of the mouse brain m^6^A-seq data with the mouse synaptic proteome revealed that 76.8% of mouse postsynaptic genes and 30% of presynaptic genes were detected with m^6^A RNA methylation [35]. In addition to the resistance of decay in the developmental- and apoptosis-associated genes during the cell fate conversion from GCPs to CGCs, we also recognized a set of synapse-related genes that are highly enriched in the abnormally spliced genes when m^6^A is depleted in the cerebellum. *Grin1* had been reported to be associated with CGC survival and contribute to the survival-promoting effect of NMDA receptors in cultured CGCs [64]. In this study, we demonstrated that the altered splicing of *Grin1* induced by m^6^A depletion resulted in excessive calcium influx and finally caused the apoptosis of the CGCs. The regulatory function of METTL3-mediated m^6^A in alternative splicing of synapse-associated genes implied its pivotal role in neuronal survival, connection, and maturation.

Although it has been reported that cytoplasmic translocation of METTL3 alone could promote protein translation independent of its m^6^A activity [65], our current study demonstrated that m^6^A depletion induced by *Mettl3* inactivation accounts for both the elevated RNA stability of development- and apoptosis-associated genes (such as *Atoh1* and *Dapk1*) and the altered splicing of synapse-associated genes (such as *Grin1*), which consequently led to the severe cerebellum development defects manifested by massive apoptosis and impaired differentiation of CGCs in the knockout mouse model. Thus, it is highly unlikely that the functional role of *Mettl3* in the mouse cerebellum development is m^6^A independent.

Contrary to a previous report showing that METTL3 is widely expressed in various regions of the mammalian brain [35], we detected relatively weak METTL3 signals in the proliferating GCPs in the outer EGL of the developing mouse cerebellum by Immunohistochemistry (IHC) (S4B Fig), which might be a likely reason to explain why the proliferation of the GCPs was not substantially affected by depletion of METTL3. However, we detected dramatically elevated expression of METTL3 when GCPs differentiated into the newborn CGCs in the inner layer of EGL (S4B Fig). We highly speculate that enhanced expression of METTL3 is a pivotal event during the differentiation of CGCs.

Taken together, we conclude that METTL3 is an essential regulator of cerebellar neurogenesis and development, and the transcriptome-wide gene expression dysregulation induced by m^6^A depletion in the absence of METTL3 finally results in CGCs apoptosis and cerebellum hypoplasia (Fig 6G).

## Materials and methods

### Ethics statement

The study was approved by the Research Ethic Committee in the Institute of Zoology, Chinese Academy of Sciences in Beijing, China (Approval number: IOZ20150076).

### Animals

Mouse strains used in this study were B6D2F1 (C57BL/6×DBA2), C57BL/6, and DBA2. B6D2F1 and DBA2 mice were purchased from Beijing Vital River Laboratory Animal Center. C57BL/6 *Nestin-Cre* mice were purchased from the Jackson Laboratory. All mice were housed under specific pathogen-free (SPF)-grade conditions in the animal facilities of the Institute of Zoology, Chinese Academy of Sciences.

### Generation of *Mettl3*^*flox/flox*^; *Nestin-Cre* mice

The T7-Cas9 plasmid and the T7-sgRNA backbone used in this study were from Qi Zhou’s lab [66]. T7-sgRNA backbone harbored a T7 promoter followed by two reverse orientated Bbsl restriction sites, which was followed by a small guide RNA scaffold. The T7-sgRNA plasmid construction was performed as previously described [66]. The annealed sgRNA oligonucleotides synthesized from Beijing Genomics Institute (BGI) have two different overhangs that are complementary with the corresponding sticky ends of Bbsl-digested T7-gRNA vector to ensure directional cloning. Oligonucleotides used for CRISPR-Cas9 system-assisted homologous recombination through embryo injection were synthesized in Integrated DNA Technologies. All oligonucleotides were listed in S4 Table.

The sgRNAs and Cas9 mRNA were transcribed by the HiScribe T7 *In Vitro* Transcription Kit (New England Biolabs) using endonuclease linearized DNA templates according to the manufacturer’s instructions. Cas9 mRNA was capped with the m7G (5′) ppp (5′) G RNA Cap Structure Analog (New England Biolabs) by T7 RNA transcriptase. RNAs were dissolved in DEPC-H_2_O (New England Biolabs) and used for intracytoplasmic RNA microinjection following previously reported procedures [66]. Briefly, one-cell-stage embryos were collected from female B6D2F1 mice at 0.5 day post-coitum (dpc). Each embryo was microinjected with 25 ng/μL L-sgRNA, 25 ng/μL R-sgRNA, 100 ng/μL Cas9 mRNA, 50 ng/μL L-oligo DNA, and 50 ng/μL R-oligo DNA into the cytoplasm. Injected embryos were subsequently implanted into the oviducts of pseudopregnant B6D2F1 female mice. Full-term pups were obtained by natural labor at 19.5 dpc.

All pups were genotyped and two *Mettl3*^*flox/+*^ founder mice were obtained. The viripotent *Mettl3*^*flox/+*^ founder mice were self-bred or mated with *Nestin-Cre* mice to generate *Mettl3*^*flox/flox*^ and *Mettl3*^*flox/+*^; *Nestin-Cre* mice, respectively. The *Mettl3*^*flox/flox*^ and *Mettl3*^*flox/+*^; *Nestin-Cre* mice were interbred to generate *Mettl3*^*flox/+*^; *Nestin-Cre*, *Mettl3*^*flox/flox*^, *Mettl3*^*flox/+*^, and *Mettl3*^*flox/flox*^; *Nestin-Cre* mice.

### Genotyping

Genomic DNA was extracted using the Mouse Direct PCR Kit (Bimake). Briefly, mouse tissues were mixed with 100 μL Buffer L and 2 μL Protease Plus, and incubated at 55 °C for 30 min, then 100 °C for 5 min according to the manufacturer’s instructions.

First, all mouse pups were genotyped to identify the insertion of loxP sites in *Mettl3* using tail DNA extract. The length of the PCR product was 222 bp/182 bp (loxP/wt) in *Mettl3 intron 1* with primers F1/R1 and 335 bp/295 bp (loxP/wt) in *Mettl3 intron 4* with primers F2/R2. Pups with loxP inserted both in *Mettl3* intron 1 and intron 4 in the same allele of *Mettl3* were identified as *Mettl3*^*flox/+*^. Pups with loxP inserted both in *Mettl3* intron 1 and intron 4 in both alleles of *Mettl3* were identified as *Mettl3*^*flox/flox*^. The PCR product for *Nestin-Cre* was 410 bp with primers Nestin-Cre-F/Nestin-Cre-R. Primers F1/R2 were used to further confirm *Mettl3* deletion in the brain, producing PCR product with 318 bp/2,554 bp (cKO/wt) in length. All sequences of primers for genotyping are listed in S5 Table.

### Western blot

Mouse brain tissues were harvested after cervical dislocation and were homogenized either by grinding into homogenous powder using mortar and pestle cooled in liquid-nitrogen bath or by mechanical homogenizer using beating beads. Pulverized tissue samples were lysed in RIPA buffer (0.5% NP-40, 50 mM Tris-Cl, pH 8, 150 mM NaCl, 1 mM EDTA) supplemented with proteinase and phosphatase inhibitors. The lysate was centrifuged at 12,000 *g* at 4 °C for 20 min to remove cell debris. Pierce Coomassie Plus (Bradford) assay kit (Thermo) was used to determine the protein concentration. The fraction (50–100 μg) was separated by 10% SDS-PAGE and analyzed by immunoblotting with corresponding antibodies, anti-Mettl3 (1:1,000, Abcam, ab195352), anti-Mettl14 (1:1,000, Atlas Antibodies, HPA038002), and anti-γ-ACTIN (1:3,000, Santa Cruz, SC65638).

### Tail suspension test

Tail suspension test was performed as described by Pierre-Olivier Frappart [36]. Mice at P14 were hung by the tail. Postures and movements of the mice were observed.

### Open field test

The assay was performed as previously described [67] using P14 Ctrl and cKO mice. The apparatus was a square-shaped arena (750 × 750 mm^2^, length × width), which was divided into two concentric squares, the central field (325 × 325 mm^2^ arena) and the peripheral field, and was illuminated evenly at 15 lux. Animals were placed in the center of the arena, and their locomotion was recorded by a camera hung over the arena. A 10-min-long video was analyzed by the TopScan software. Locations of the animals in the area were recorded at the speed of one image per s. The frequency crossing the border, latency in the central field, total moving distance, and velocity of mice recorded in the apparatus were analyzed.

### MRI and stereological measurements

All mice for MRI were anesthetized with 10% chloralic hydras (Sigma) by intraperitoneal injection. Mice were fixed on the scanning coil with a self-made scaffold. Body temperature was maintained by circulating water through warming pads. MRI was conducted using a Bruker 7.0 T MRI equipment (Bruker Medical Systems, CLinScan) and was performed at P14 for both Ctrl and cKO mice. Both the sagittal section and transverse section were scanned. For image acquisition, rapid acquisition relaxation enhanced (RARE) sequence was used. The MRI parameters included T2W repetition time = 2,500 ms, T2W echo time = 60 ms, field of view = 12 × 12 mm, matrix = 240 × 240 pixels, resolution = 50 × 50 μm, and slice thickness = 0.6 mm. MRI images acquired were processed by ImageJ (https://imagej.nih.gov/ij/) to calculate the area of the whole brain section or the cerebellar region. To be brief, the outline of the whole brain or the cerebellum in the image was drawn out as region of interest (ROI); the number of pixels contained within the ROI was calculated by the software. As the resolution is 50 × 50 μm, each pixel represents an area of 2.5 × 10^−3^ mm^2^. Three repeats were conducted for each calculation.

### BrdU labeling

In order to examine the proliferation activity of GCPs in the cerebellar EGL, we injected P7 mice with BrdU (Sigma) intraperitoneally at a dose of 50 μg BrdU/g body weight [36]. Mice were humanely killed 2 h later for immunofluorescence staining. To investigate the generation and migration of CGCs, BrdU-injected mice were kept for another 2 d until they were humanely killed at P9 for staining.

### Macromorphological observation

To compare the gross appearance and histological organization of the cerebellums of both Ctrl and cKO mice, mice at P14 were humanely killed by breaking the neck. The whole brain or cerebellum was dissected, weighed, or fixed with 4% paraformaldehyde and photographed.

### Hematoxylin and eosin analysis

Dissected brains were cut into sagittal blocks and fixed with 4% paraformaldehyde followed by dehydration (70%, 80%, 90%, 100% ethanol) and paraffin embedding. Sections 3 μm in thickness were cut from the paraffin-embedded tissue blocks with a Leica slicing machine (Leica Biosystems) and mounted on poly-D-lysine coated glass slices (Zhong Shan Golding Bridge Biotechnology). Slices were heated at 65 °C for 2 h and then immersed in xylene to remove paraffin. After a series of rehydration processes (100%, 100%, 90%, 80%, 70% ethanol), slices were stained with hematoxylin and eosin (HE) using standard methods and imaged with a Leica Aperio VERSA 8 microscope (Leica Biosystems).

### Immunohistochemical analysis

For immunohistochemical staining, rehydrated sections or coverslips were incubated with primary antibodies overnight. Primary antibodies used in this study were for Mettl3 (ab195352, 1:250, Abcam), neuronal nuclei (NeuN, MAB377, 1:500, Millipore), calbindin D-28K (C9848, 1:2,000, Sigma), and glial fibrillary acid protein (GFAP, Z033429, 1:1,000, Dako). Appropriate Horseradish peroxidase conjugated secondary antibodies (anti-Mouse IgG and anti-Rabbit IgG, Vector laboratories) were used according to manufacturer’s recommendations. A 3, 3′-diaminobenzidine kit (DAB, Vector laboratories) was used for color developing. Images were obtained using standard methods and imaged with a Leica Aperio VERSA 8 microscope (Leica Biosystems). Immunohistochemical images were analyzed with Image-Pro Plus (Media Cybernetics). At least three repeats were conducted for each calculation.

### Immunofluorescence analysis

Immunofluorescence staining was performed as previously described, with a few modifications [68]. Briefly, rehydrated sections or coverslips were incubated with primary antibodies overnight. Primary antibodies used in this study were for BrdU (C8434, 1:500, Sigma), Ki67 (ab15580, 1:500, Abcam), Cleaved Caspase-3 (9661s, 1:200, Cell Signaling Technology), Phospho-Histone H3 (3377s, 1:500, Ser10, Cell Signaling Technology), Nestin (MAB353, 1:1,000, Millipore,), Sox2 (SC-17320, 1:1,000, Santa Cruz), and Mettl3 (ab195352, 1:1,000, Abcam). Appropriate fluorophore (FITC, Cy3, and Cy5) conjugated secondary antibodies (Jackson ImmunoResearch, 1:1,000) were used according to the manufacturer’s recommendations. Sections were counterstained with DAPI (Invitrogen) and mounted on slices with Dako Fluorescence Mounting Medium (Dako). Fluorescent images were obtained using a Carl Zeiss LSM 780 confocal system. For quantification of Ki67 and Phospho-Histone H3 positive cell ratio, we used three pairs of mice at P7 and quantified three sections of the cerebellum vermis for each Ctrl and cKO mouse, respectively. We lined out the EGL region manually, and the total numbers of Ki67^+^-, Phospho-Histone H3- and DAPI-positive cells in the EGL region were analyzed with Columbus Image Analyzing System (Perkin Elmer). For the quantification in the assays of long-term BrdU labelling, TUNEL, and Cleaved-Caspase 3 staining, the positive cells in the whole section of the cerebellum vermis were quantified. Three pairs of mice and three sections for each of Ctrl and cKO mouse were used for quantification.

### TUNEL analysis

The TUNEL assay was performed using the DeadEnd Fluorometric TUNEL System (Promega) according to the manufacturer’s instruction. Briefly, sections were permeabilized by proteinase K and labeled with rTdT reaction mix for 1 h at 37 °C; the reaction was stopped by 2 × SSC. Sections were counterstained by PI (Invitrogen) and mounted on slices with Dako Fluorescence Mounting Medium (Dako). Images were obtained using a Carl Zeiss LSM 780 confocal system. Fluorescent images were analyzed with Columbus Image Analyzing System (Perkin Elmer). At least three repeats were conducted for each calculation.

### RNA isolation

Total RNA from mouse cerebellums was extracted using TRIzol reagent (Invitrogen). mRNAs were purified with Dynabeads mRNA Purification Kit (Thermo) according to the manufacturer’s manual, followed by DNase I (M6101, Promega) treatment to remove genomic DNA. RNA concentration was measured using Nanodrop 1000 (Thermo). For quality control, only samples with OD 260/280 nm ratio more than 2 and 260/230 nm values in the range of 2.0–2.2 were used for subsequent experiments. The integrity of RNAs was checked by RNA gel electrophoresis, and only total RNAs having a 28S band twice as bright as the 18S band were used for further study. Two sets of samples were collected as biological replicates.

### UHPLC-MS/MS analysis of mononucleotides

A total of 400 ng RNA was mixed with 0.1 U Nuclease P1 (Sigma) and 1.0 U calf intestinal phosphatase (New England Biolabs) in the final reaction volume of 50 μL, adjusted with water and incubated at 37 °C overnight. The digested RNA solutions were filtered by ultrafiltration tubes (MW cutoff: 3 kDa, Pall, Port Washington), then subjected to UHPLC-MS/MS analysis for detection of m^6^A. The UHPLC-MS/MS analysis was performed with an Agilent 1290 UHPLC system coupled with a G6410B triple quadrupole mass spectrometer (Agilent Technologies). A CAPCELL PAK C18 column (100 × 2.1 mm I.D., 3 μm particle size, SHISEIDO) was used for UHPLC separation of mononucleotides. UHPLC separation parameters were used as follows: 0–6.5 min, 5.0% B; 6.6–11.0 min, 20.0% B; 11.1–16.0 min, 100% B; 16.1–24 min, 5.0% B. Solvent A was an aqueous solution of 0.1% formic acid, and solvent B was 100% methanol. The mass spectrometer was operated in the positive ion mode. A multiple reaction monitoring (MRM) mode was adopted, using m/z 282→150 for m^6^A (collision energy, 15 eV) and m/z 268→136 for A (10 eV). The injection volume for each sample was 5 μL, and the amount of m^6^A and A was calibrated by standard curves. Nitrogen was used for nebulizing and desolvation gas of MS detection. The nebulization gas was set at 40 psi, the flow rate of desolvation gas was 9 L/min, and the source temperature was set at 300 °C. Capillary voltage was set at 3,500 V. High purity nitrogen (99.999%) was used as collision gas. Each sample was analyzed at least three times.

### Methylated RNA immunoprecipitation

Methylated RNA immunoprecipitation (MeRIP) was performed as previously described [69]. Briefly, purified mRNA was randomly fragmented to size around 100 nucleotides using Ambion RNA fragmentation reagents and then subjected to immunoprecipitation (IP) with anti-m^6^A antibody (202003, Synaptic Systems) and protein-A magnetic beads (88845, Pierce) in MeRIP buffer (150 mM NaCl, 10 mM Tris-HCl, pH 7.4, 0.1% NP-40) supplemented with RNase inhibitor. m^6^A-containing mRNA fragments were eluted with m^6^A in MeRIP buffer and were purified using TRIzol reagent. For MeRIP-seq, two sets of samples were collected for duplicated biological repeats. For MeRIP-qRT-PCR, the same procedures were carried out except that the purified mRNA was fragmented into about 200 nucleotides; three biological repeats were conducted.

### RNA-seq and m^6^A-seq

Poly (A) RNA from 1 mg total RNA or purified mRNA and purified m^6^A-containing fragments were used to generate the cDNA libraries, respectively, according to TruSeq RNA Sample Prep Kit protocol. All samples were sequenced by Illumina HiSeq-3000 with paired-end 101-bp read length. About 100 million and 150 million 2*101 paired-end reads in cKO and Ctrl mice were obtained, respectively.

### Bioinformatic analysis

Raw RNA-seq reads were mapped to the mouse reference genome (mm10) using HISAT2 (Version 2.0.4) with parameters “—novel-splicesite-infile” [70]. StringTie (Version 1.3.1c) was used to construct full-length transcript assembly and estimate transcript abundance with default parameters [71]. Transcripts were filtered by TACO (Version 0.5.1) [72] with default parameters. Differentially expressed genes between the Ctrl and cKO mice at P7 and P14 were identified using edgeR (Version 3.10.0) [73] with fold change ≥ 1.5 and adjusted *p*-value ≤ 0.1 (Benjamini–Hochberg multiple testing correction) as the thresholds.

To identify decreased or increased m^6^A peaks in Ctrl and cKO m^6^A-seq against RNA-seq, two replicates for each group were used for differential peak analysis. The sequences were normalized according to total library size prior to peak calling (see S6 Table for normalization factors). A peak calling prioritization pipeline (PePr, version 1.1.18) was used to identify differential binding sites of m^6^A caused by *Mettl3* conditional knockout [74]. A 200-bp sliding window approach and intergroup normalization were performed for m^6^A peak calling after removing PCR duplication, and a *p*-value threshold of 1e-5 (Wald test) was reported. The m^6^A peaks were annotated by bedtools (version 2.25.0) [75]. The enriched sequence motifs among m^6^A peaks were identified by HOMER (version 4.7) [76] with motif lengths of 5 nt, 6 nt, 7 nt, and 8 nt, respectively.

Gene Ontology biological processes and Reactome gene sets enrichment analysis were carried out using Metascape with *p*-value < 0.01 (Banjamini–Hochberg multiple testing correction), with the expressed genes (FPKM > 0.1) in Ctrl mouse cerebellums as the background [77]. For the enriched terms, Kappa scores were used as the similarity metric when performing hierarchical clustering, and then subtrees with similarity > 0.3 were considered as a cluster [77]. Enriched terms were selected according to the Metascape cluster results.

To analyze alternative splicing, the PSI method provided by Schafer and colleagues was used [78]. Briefly, annotations on an exon sequence were created through DEXseq (Version 1.20.2) [79], based on assembled transcripts. The PSI ratios for all exon parts were estimated by modifying the code provided by Schafer and colleagues [77]. PSI comparison supported by at least 10 inclusion and exclusion reads was considered for further analysis. Exons with ΔPSI ≥ 10% in Ctrl mice compared to cKO mice were considered as ASEs. ASEs overlapped with m^6^A-modified genes in Ctrl were further analyzed to uncover the target exons and their splicing pattern affected by METTL3.

### Cell cultures

For establishment of the neural stem cell (NSC) lines, neonatal mouse brains were cut into small pieces by ophthalmic scissors and digested with 0.25% Typsin-EDTA (Gibco) for 15 min in 37 °C, 5% CO_2_. The dissociated cell suspension was plated on Matrigel (BD Biosciences) coated 6-well plates. The basal culture medium contained Neurobasal Medium (Gibco) and DMEM/F12 Medium (Gibco) at a ratio of 1:1, plus 0.5% N-2 Supplement (Gibco), 1% B-27 Serum-Free Supplement (Gibco), 2 mM GlutaMAX-I (Gibco), bFGF (20 ng/mL, R & D systems), and EGF (20 ng/mL, R & D systems). For immunofluorescent staining, NSCs were plated on coverslips coated with poly-L-ornithine (Sigma) and Matrigel (BD Biosciences), successively. HeLa cells were cultured in 37 °C, 5% CO_2_ in DMEM Medium (Gibco) plus 10% FBS (Gibco), 2 mM GlutaMAX-I (Gibco), and 0.01 mM beta-mercapto-thanol (beta-ME, Gibco).

CGCs were isolated from P7–P8 mouse pups as previously described [80]. To be brief, cerebellums were dissected and washed by HBSS (Gibco) and dissociated with 0.25% Typsin-EDTA (Gibco). Cells were suspended and subjected to Percoll (GE Healthcare) gradient (30% and 65% Percoll) separation. The astroglia and other heavier cells were separated by pre-plating on a poly-D-lysine (Sigma) coated dish for 20 min in a 5% CO_2_, 37 °C incubator. Purified CGCs were plated on poly-D-lysine coated plates and cultured in Neurobasal Medium (Gibco) containing 2% B-27 Serum-Free Supplement (Gibco), 2 mM GlutaMAX-I (Gibco), and 15 μM glutamate (Gibco) in a 5% CO_2_, 37 °C incubator. After 20 h, 10 μM Cytosine arabinoside (Ara-C, Sigma) was added to arrest the proliferation of nonneuronal cells.

### Mouse CGC electroporation

The plasmids Pmax-mCherry, Pmax-*Grin1*_*001*_-T2A-mCherry, or Pmax-*Grin1*_*011*_-T2A-mCherry were constructed using the Pmax-GFP backbone (Lonza). Approximately 5 × 10^6^ mouse CGCs were resuspended in 100 μL P3 Solution from the Lonza Nucleofector P3 Primary Cell 4D X Kit (Lonza). Additionally, 10 μg of the appropriate plasmid was added to the cell suspension. The cell suspension was then placed in the cuvette and electroporated using a Lonza Nucleofector 4D device (Lonza) under the presupposed program CD150. Cells were then resuspended and cultured in CGC culture medium.

### Calcium imaging and measurement

Electroporated CGCs were plated on a 20 mm confocal dish (Nest) precoated with poly-D-lysine. Culture medium of 3 DIV neurons was replaced by 2 mL of Locke’s solution containing 154 mM NaCl, 5 mM KCl, 3.6 mM NaHCO_3_, 5 mM HEPES, 1.5 mM CaCl_2_, 1.2 mM MgCl_2_, and 5.6 mM glucose, pH 7.4, and incubated for 10 min at room temperature. Cells were subsequently incubated for 20 min in 2 mL of Locke’s solution containing 10 pM Fluo-4 (Thermo) and then washed with 2 mL of Locke’s solution for 10 min before starting the experiment. All measurements were made at room temperature (25 °C). Fluo-4 (green) and mCherry (indicating the expression of appropriate plasmid, red) fluorescence were imaged with an inverted Perkin-Elmer microscope. Images were digitized in an image processor connected to a computer equipped with VOLOCITY (version 6.0) software. The formula [Ca^2+^]_i_ = Kd_Fluo-4_ × [F − F_min_/ F_max_ − F] [81] was used to calculate the intracellular calcium concentration of mouse CGCs. F_max_ and F_min_ were obtained by perfusing cells with a salt solution containing 10 mM CaCl_2_, and subsequently with a Ca^2+^-free salt solution containing 10 mM EGTA (Calcium Calibration Buffer Kit, Thermo).

### CCK8 assay

Electroporated CGCs were seeded on 48 multi-wells (Corning) coated with poly-D-lysine. At DIV 1, DIV 3, and DIV 5, cells were incubated with CGC culture medium containing 1 × CCK8 (Solarbio) for 4 h in a 5% CO_2_, 37 °C incubator. The absorbance was measured using a scanning multi-well spectrophotometer (ELISA reader) at a wavelength of 450 nm. The survival rates of CGCs were calculated as OD450_DIV3_/OD450_DIV1_ × 100% or OD450_DIV5_/OD450_DIV1_ × 100%.

### RNA half-life detection

NSCs were plated on 6-well plates, 5 × 10^5^ per well, for 2 d, then cells were treated with actinomycin-D (10 μg/mL, Sigma) and collected for qRT-PCR analysis 3 h, 6 h, 9 h, or 12 h later. The half-life was calculated according to the following equation: ln (C_i_/C_0_) = −kt_i_, where k is degradation rate, C_i_ is the mRNA value at time i, and t_i_ is the time interval in hours. Three repeats were conducted for each calculation.

### qRT-PCR and semiquantitative RT-PCR

In the qRT-PCR experiments, 1 μg of total RNA treated with DNase I was reversely transcribed into cDNA by the Reverse Transcription System (Promega). SYBR Green PCR Master Mix (Toyobo) was used in all qRT-PCR experiments. The relative fold expression changes of genes were calculated using the 2^-ΔΔCt^ method, with *Actb* as internal control. Semiquantitative RT-PCR products were amplified for 30 cycles. Primers used in these experiments are listed in S7 Table.

### MiniGene splicing assay

For miniGene-based splicing analysis of exon 19 in *Lrp8* and exon 21 in *Grin1*, a fragment containing either wild-type (miniGene) or mutated m^6^A motif (miniGene-M) was inserted into the pSpliceExpress reporter vector (Addgene). For *Lrp8*, two m^6^A motifs (GAACC) were mutated to GATCC. For *Grin1*, one m^6^A motif (AGACA) was mutated to AGTCA. MiniGene or miniGene-M was transfected into HeLa cells with control siRNA (siControl) or siRNAs for knockdown of *Mettl3* (si*Mettl3*) using Lipofectamine 3000 (Invitrogen), respectively. Forty-eight hours after transfection, total RNA was isolated and reverse transcription was carried out to produce the cDNA. Then, cDNA was used as template for alternative splicing change detection through semiquantitative RT-PCR. AlphaView was used to analyze the density of bands on agar gels. Ratios of exon exclusion were quantified based on the density of bands. All the primers and siRNAs used for this assay are listed in S8 Table.

### Statistical analysis

All statistical analyses (unless stated otherwise) were performed using the R package for Statistical Computing. For experimental data quantification, Student *t* test was applied using GraphPad Prism 6 software, and the error bar was shown based on SEM (unless stated otherwise). *P* value < 0.05 was considered statistically significant.

## Supporting information

S1 Fig Generation of *Nestin-Cre* mediated *Mettl3* conditional knockout mice. (A) Mettl3 immunohistochemical analysis for both Ctrl and cKO mouse brains at P14. Scale bar, 1 mm. (B) Diagram illustrating the procedure of generating *Nestin-Cre* mediated *Mettl3* conditional knockout mice. (C) Genotyping using tail-tip DNA for the *Mettl3*^*+/+*^, *Nestin-Cre*, *Mettl3*^*flox/+*^, *Mettl3*^*flox/flox*^, *Mettl3*^*flox/+*^;*Nestin-Cre*, and *Mettl3*^*flox/flox*^; *Nestin-Cre* mice. (D) Sanger sequencing of the loxP locus of *Mettl3* of P7 brain extract DNA of the *Mettl3*^*flox/flox*^;*Nestin-Cre* mice. (E) Sanger sequencing for the loxP locus inserted in intron 1 of *Mettl3* using tail-tip DNA of newborn *Mettl3*^*flox/flox*^;*Nestin-Cre* mice. (F) Sanger sequencing for the loxP locus inserted in intron 4 of *Mettl3* using tail-tip DNA of newborn *Mettl3*^*flox/flox*^;*Nestin-Cre* mice. (G) PCR confirms the cKO of *Mettl3* exons 2–4 in the brain of *Mettl3*^*flox/flox*^;*Nestin-Cre* mice. (H) Western blot from brain and liver extracts in both Ctrl and cKO mice at P7 confirms the depletion of METTL3 protein in *Mettl3*^*flox/flox*^;*Nestin-Cre* mice brains. γ-ACTIN, loading control; Ctrl, control; cKO, *Mettl3* conditional knockout; loxP, locus of X-over P1; flox, a allele with two forward loxp sequences; *Mettl3*, methyltransferase-like 3; *Mettl3*^*flox/+*^, *Mettl3* gene with a flox allele and a wild-type (+) allele; *Mettl3*^*flox/flox*^, *Mettl3* gene with two flox alleles; *Nestin-Cre*, Cre recombinase expression driven by *Nestin* promoter.
(TIF)

S2 Fig Neuronal specific inactivation of *Mettl3* in mice causes growth retardation and neonatal death. (A) Body size comparison of the Ctrl and cKO mice at P14. (B–D) Open field test shows the representative activity traces (B), time spent in the central region (C), and bouts from the central field to the peripheral field (D) for both Ctrl and cKO mice at P14. (E) MRI analysis for the whole brain transverse section of the Ctrl and cKO mice at P14. Scale bar, 1 mm. (F) UHPLC-MS/MS quantification of m^6^A levels in mRNAs isolated from the Ctrl and cKO mouse brain at P7 and P14. Further information about this figure can be found in S1 Data. The data were represented as means ± SEM. Three technical measurements from two biological replicates were performed. **p-value < 0.01, ***p-value < 0.001, Student *t* test. CF, central field; cKO, *Mettl3* conditional knockout; Ctrl, control; Mettl3, methyltransferase-like 3; MRI, magnetic resonance imaging; PF, peripheral field; SEM, standard error of the mean; UHPLC-MS/MS, ultra high-pressure liquid chromatography tandem-mass spectrometry.
(TIF)

S3 Fig Difference of m^6^A enzyme expression and m^6^A level between cerebellum and other brain regions of wild-type mice. (A) Western blot comparing the expression of METTL3 and METTL14 between the cerebellum and cerebral cortex of wild-type mice. (B) UHPLC-MS/MS quantification of m^6^A levels in mRNAs isolated from the cerebellum and brain regions minus the cerebellum. Data related to this figure are shown in S1 Data. Data shown are means ± SEM. Three technical measurements from two biological replicates were performed. **p-value < 0.01, Student *t* test. γ-ACTIN, loading control; METTL3, methyltransferase-like 3; METTL14, methyltransferase-like 14; m^6^A, *N*^6^-methyladenosine; SEM, standard error of the mean; UHPLC-MS/MS, ultra high-pressure liquid chromatography tandem-mass spectrometry.
(TIF)

S4 Fig Histological examination of cerebellums from the Ctrl and cKO mice. (A–C) Immunohistochemical analysis of Mettl3 in the cerebellum at E16.5, P7, and P14. Scale bar, 200 μm. (D) HE staining and immunohistochemical analysis of Ctrl and cKO cerebellums at P7. Scale bar, 200 μm. cKO, *Mettl3* conditional knockout; Ctrl, control; EGL, external granular layer; HE, hematoxylin and eosin; IGL, internal granular layer; ML, molecular layer; PCL, Purkinje cell layer.
(TIF)

S5 Fig Neuronal specific deletion of *Mettl3* in mice induces apoptosis of newborn granule cells. (A) Immunofluorescent staining of PH3 (green) and DAPI (blue) in cerebellums of Ctrl and cKO mice at P7. Scale bar for the left panels, 200 μm. Scale bar for the right panels, 25 μm. (B) Proportion of PH3^+^/DAPI^+^ cells in the EGL of Ctrl and cKO mice at P7. Further information about this figure can be found in S1 Data. The data were represented as means ± SEM; *n* = 3 for each group. *p-value < 0.05, Student *t* test. (C) High magnificent HE staining images of EGL cells from Ctrl and cKO mice at P7. Black triangles indicate cells with karyorrhexis and karyopyknosis. Scale bar, 200 μm. cKO, *Mettl3* conditional knockout; Ctrl, control; EGL, external granular layer; HE, hematoxylin and eosin; PH3, phosphorylated H3; SEM, standard error of the mean.
(TIF)

S6 Fig m^6^A depletion induces dramatic gene expression change in the cerebellum at P14. (A) The most enriched sequence motif of m^6^A peaks in RNAs from P14 mouse cerebellum. (B) Distribution of m^6^A peaks along the 5′UTR, CDS, and 3′UTR regions of total cerebellar mRNAs from P14 Ctrl mice after normalized with length. (C) Venn diagram representing the relationships between cerebellar expressed genes and m^6^A modification. Green circle represents all expressed genes in P14 Ctrl mouse cerebellum, brown circle represents genes with m^6^A modifications in P14 Ctrl mouse cerebellum, red circle represents genes with up-regulated expression in P14 *Mettl3* cKO mouse cerebellum as compared to the Ctrl, and blue circle represents genes with down-regulated expression in P14 *Mettl3* cKO mouse cerebellum as compared to the Ctrl. Numbers represent the counts of genes in each group. (D) Significantly enriched (*p*-value ≤ 0.01, Banjamini–Hochberg multiple testing correction) GO terms of genes with up-regulated expression in P14 cKO cerebellums and with m^6^A peaks. (E) qRT-PCR results confirmed the up-regulated expression in P14 cKO cerebellums of selected genes. The major functions of detected genes are shown under the lines. Further information about this figure can be found in S1 Data. The data were represented as means ± SEM; *n* = 3. *p-value < 0.05, **p-value < 0.01, ***p-value < 0.001, Student *t* test. CDS, coding sequence; cKO, *Mettl3* conditional knockout; GO, gene ontology *Mettl3*, methyltransferase-like 3; m^6^A, *N*^*6*^-methyladenosine; qRT-PCR, quantitative reverse transcription-polymerase chain reaction; SEM, standard error of the mean.
(TIF)

S7 Fig GO enrichment analysis of up-regulated and down-regulated genes in cKO cerebellum as compared with the Ctrl. (A–D) Enriched GO terms (*p*-value ≤ 0.01, Benjamini–Hochberg multiple testing correction) of differentially up-regulated genes in P7 cKO (A), down-regulated genes in P7 cKO (B), up-regulated genes in P14 cKO (C), and down-regulated genes in P14 cKO (D) as compared with the paired Ctrl. cKO, *Mettl3* conditional knockout; GO, gene ontology.
(TIF)

S8 Fig Depletion of m^6^A in the differentially expressed genes and alternatively spliced genes in the cKO cerebellums. (A–B) The m^6^A peak plots of the differentially expressed genes (A) and the alternatively spliced genes (B) in *Mettl3* cKO mice. The abundance of normalized m^6^A-seq data and normalized RNA-seq data are shown in red and yellow, respectively. (C) MeRIP qRT-PCR results confirmed the depletion of m^6^A modification in the differentially expressed genes and alternatively spliced genes in the cKO cerebellums. Further information about this figure can be found in S1 Data. The data were represented as means ± SEM. *p-value < 0.05, **p-value < 0.01, ***p-value < 0.001. cKO, *Mettl3* conditional knockout; m^6^A, *N*^*6*^-methyladenosine; m^6^A-seq, m^6^A sequencing; MeRIP, methylated RNA immunoprecipitation; *Mettl3*, methyltransferase-like 3; qRT-PCR, quantitative reverse transcription polymerase chain reaction; RNA-seq, RNA-sequencing; SEM, standard error of the mean.
(TIF)

S9 Fig *Mettl3* mediated m^6^A regulates alternative splicing at P14. (A) Genes producing exon-excluded transcripts in P14 cKO cerebellums as compared with the Ctrl. (B) Enriched pathways of genes producing exon-excluded transcripts in P14 cKO cerebellums as compared with the Ctrl. (C) Production of exon-excluded transcripts of selected genes in P14 cKO cerebellums. Left, IGV tracks displaying the RNA-seq reads coverage in P7 Ctrl (blue) and cKO (red) cerebellum; the excluded exons are shaded with pink columns. Y-axis: normalized reads counts. Middle, calculated exon-exclusion percentage in P7 cKO cerebellums by the PSI value. Right, semiquantitative PCR detection of exon-excluded transcripts in P7 cKO cerebellums. Further information about this figure can be found in S1 Data. The data were represented as means ± SEM. **p-value < 0.01, ***p-value < 0.001, Student *t* test. cKO, *Mettl3* conditional knockout; Ctrl, control; IGV, integrative genomics viewer; *Mettl3*, methyltransferase-like 3; m^6^A, *N*^*6*^-methyladenosine; PSI, percent spliced in index; RNA-seq, RNA-sequencing; SEM, standard error of the mean.
(TIF)

S10 Fig MiniGene splicing assay for *Grin1* and *Lrp8*. (A–B) The exclusion level of exon 21 of *Grin1* (A) and exon 19 in *Lrp8* (B) was validated by RT-PCR in HeLa cells transfected with (1) siControl and miniGene, (2) si*Mettl3* and miniGene, (3) siControl and miniGene-M, and (4) si*Mettl3* and miniGene-M. (C–D) MeRIP qRT-PCR results confirmed the depletion of m^6^A modification on the alternatively spliced exon of Grin1(C) and Lrp8 (D), with mutations in the predicted m^6^A modification sites. Further information about this figure can be found in S1 Data. Values and error bars in all plots represent the mean and SEM of three independent experiments by Student *t* test. *p-value < 0.05, **p-value < 0.01, ***p-value < 0.001, ****p-value < 0.0001. Grin1, glutamate ionotropic receptor NMDA type subunit 1; Lrp8, low density lipoprotein receptor-related protein 8; MeRIP, methylated RNA immunoprecipitation; miniGene-M, miniGene-Mutant; m^6^A, *N*^*6*^-methyladenosine; qRT-PCR, quantitative reverse transcription polymerase chain reaction; SEM, standard error of the mean; siControl, control siRNA; si*Mettl3*, *Mettl3* siRNA.
(TIF)

S1 Table Number of m^6^A peaks identified from the cerebellums of cKO and Ctrl mice at P7 and P14. cKO, *Mettl3* conditional knockout; Ctrl, control; m^6^A, *N*^*6*^-methyladenosine.
(XLSX)

S2 Table Differential m^6^A binding peaks identified from the cerebellums of Ctrl mice compared with cKO mice at P7. cKO, *Mettl3* conditional knockout; Ctrl, control; m^6^A, *N*^*6*^-methyladenosine.
(XLSX)

S3 Table Differential m^6^A binding peaks identified from the cerebellums of Ctrl mice compared with cKO mice at P14. cKO, *Mettl3* conditional knockout; Ctrl, control; m^6^A, *N*^*6*^-methyladenosine.
(XLSX)

S4 Table Oligonucleotides synthesized for *loxP* insertion and T7-sgRNA plasmids construction. T7-sgRNA, T7 promoter driven sgRNA expressing vector.
(XLSX)

S5 Table Primers used for genotyping by PCR. (XLSX)

S6 Table Normalization factors for different samples. (XLSX)

S7 Table Primers used for qRT-PCR and for detection of alternative splicing. qRT-PCR, quantitative reverse transcription polymerase chain reaction.
(XLSX)

S8 Table Primers and siRNAs used for miniGene-based splicing assay for *Lrp8 exon 19*. siRNA, small interfering RNA.
(XLSX)

S1 Data Raw data used for quantification in this work. (XLSX)

S1 Movie A short video capturing the movement of the cKO and Ctrl mouse. The cKO mouse showed balance disorders and altered gaits. cKO, *Mettl3* conditional knockout.
(MOV)

S2 Movie A short video capturing the tail suspension test of the Ctrl mouse. (MOV)

S3 Movie A short video capturing the tail suspension test of the cKO mouse. cKO, *Mettl3* conditional knockout.
(MOV)

## Abbreviations

AMPA: α-amino-3-hydroxy-5-methyl-4-isoxazolepropionic acid
ASE: alternatively spliced exon
BGI: Beijing Genomics Institute
BrdU: bromodeoxyuridine
CaM: calmodulin
CCK8: Cell Counting Kit-8
CGC: cerebellar granule cell
cKO: *Mettl3* conditional knockout
CREB: cAMP responsive element binding protein
Ctrl: control
D-28K: calbindin 1
dpc: day post-coitum
E16.5: embryonic day 16.5
EGL: external granular layer
FTO: fat mass and obesity-associated protein
*GAP-43*: growth associated protein 43
GCP: granule cell progenitor
GFAP: glial fibrillary acid protein
GRIN1: glutamate ionotropic receptor NMDA type subunit 1
GRIN2: glutamate ionotropic receptor NMDA type subunit 2
HDAC1: histone deacetylase 1
HE: hematoxylin and eosin
IGL: internal granular layer
IGV: integrative genomics viewer
IHC: immunohistochemistry
IP: immunoprecipitation
Ki67: antigen identified by monoclonal antibody Ki 67
m^6^A: *N*^*6*^-methyladenosine
m^6^A-seq: m^6^A sequencing
MeRIP: methylated RNA immunoprecipitation
METTL14: methyltransferase-like 14
METTL3: methyltransferase-like 3
Mettl3^*flox/+*^: *flox* inserted in either alleles of *Mettl3*
Mettl3^*flox/flox*^: *flox* inserted in both alleles of *Mettl3*
miRNA: micro RNA
ML: molecular layer
MRI: magnetic resonance imaging
MRM: multiple reaction monitoring
NeuN: neuronal nuclei
NMDA: N-methyl-D-aspartic acid
NSC: neural stem cell
P21: 21 days postpartum
PCL: Purkinje cell layer
PKMT: lysine methyltransferase
PPAR: peroxisome proliferator-activated receptor
PSI: percent spliced in index
qPCR: quantitative polymerase chain reaction
qRT-PCR: quantitative reverse transcription-polymerase chain reaction
RARE: rapid acquisition with relaxation enhancement
RNA-seq: RNA sequencing
ROI: region of interest
RT-PCR: reverse transcription polymerase chain reaction
SEM: standard error of the mean
sgRNA: small guide RNA
siControl: control siRNA
SPF: specific pathogen-free
UHPLC-MS/MS: ultra high-pressure liquid chromatography tandem-mass spectrometry
WTAP: Wilms’ tumor 1-associating protein
YTHDF1: YTH N6-methyladenosine RNA binding protein 1
