# Supplementary figures and images for: METTL3-mediated m6A modification is required for cerebellar development
Source: PLoS Biol. 2018 Jun 7;16(6):e2004880. doi: 10.1371/journal.pbio.2004880 (PMC6021109; doi:10.1371/journal.pbio.2004880)

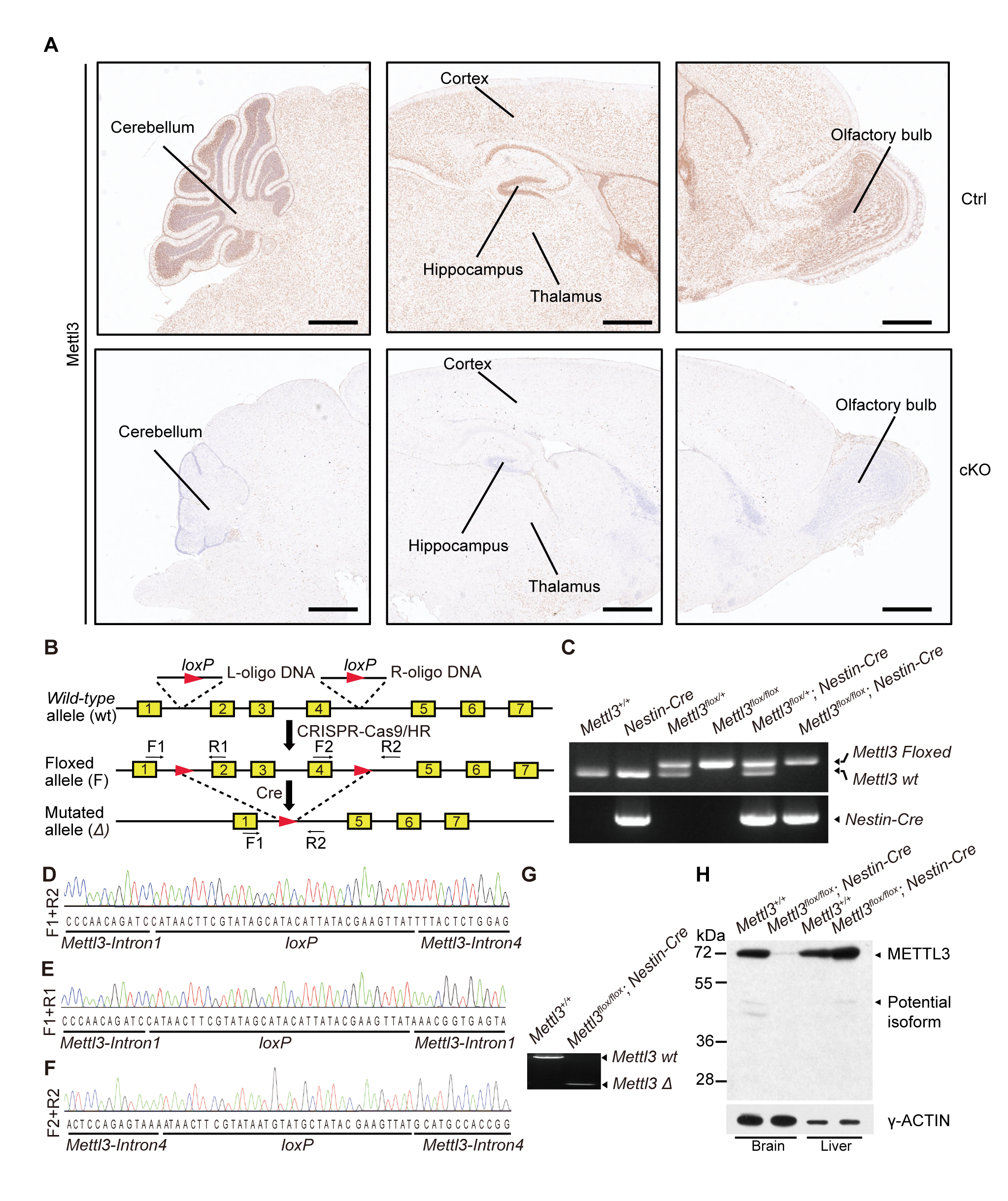

Supplement: S1 Fig — (A) Mettl3 immunohistochemical analysis for both Ctrl and cKO mouse brains at P14. Scale bar, 1 mm. (B) Diagram illustrating the procedure of generating Nestin-Cre mediated Mettl3 conditional knockout mice. (C) Genotyping using tail-tip DNA for the Mettl3+/+, Nestin-Cre, Mettl3flox/+, Mettl3flox/flox, Mettl3flox/+;Nestin-Cre, and Mettl3flox/flox; Nestin-Cre mice. (D) Sanger sequencing of the loxP locus of Mettl3 of P7 brain extract DNA of the Mettl3flox/flox;Nestin-Cre mice. (E) Sanger sequencing for the loxP locus inserted in intron 1 of Mettl3 using tail-tip DNA of newborn Mettl3flox/flox;Nestin-Cre mice. (F) Sanger sequencing for the loxP locus inserted in intron 4 of Mettl3 using tail-tip DNA of newborn Mettl3flox/flox;Nestin-Cre mice. (G) PCR confirms the cKO of Mettl3 exons 2–4 in the brain of Mettl3flox/flox;Nestin-Cre mice. (H) Western blot from brain and liver extracts in both Ctrl and cKO mice at P7 confirms the depletion of METTL3 protein in Mettl3flox/flox;Nestin-Cre mice brains. γ-ACTIN, loading control; Ctrl, control; cKO, Mettl3 conditional knockout; loxP, locus of X-over P1; flox, a allele with two forward loxp sequences; Mettl3, methyltransferase-like 3; Mettl3flox/+, Mettl3 gene with a flox allele and a wild-type (+) allele; Mettl3flox/flox, Mettl3 gene with two flox alleles; Nestin-Cre, Cre recombinase expression driven by Nestin promoter. (TIF) [file pbio.2004880.s001.tif]

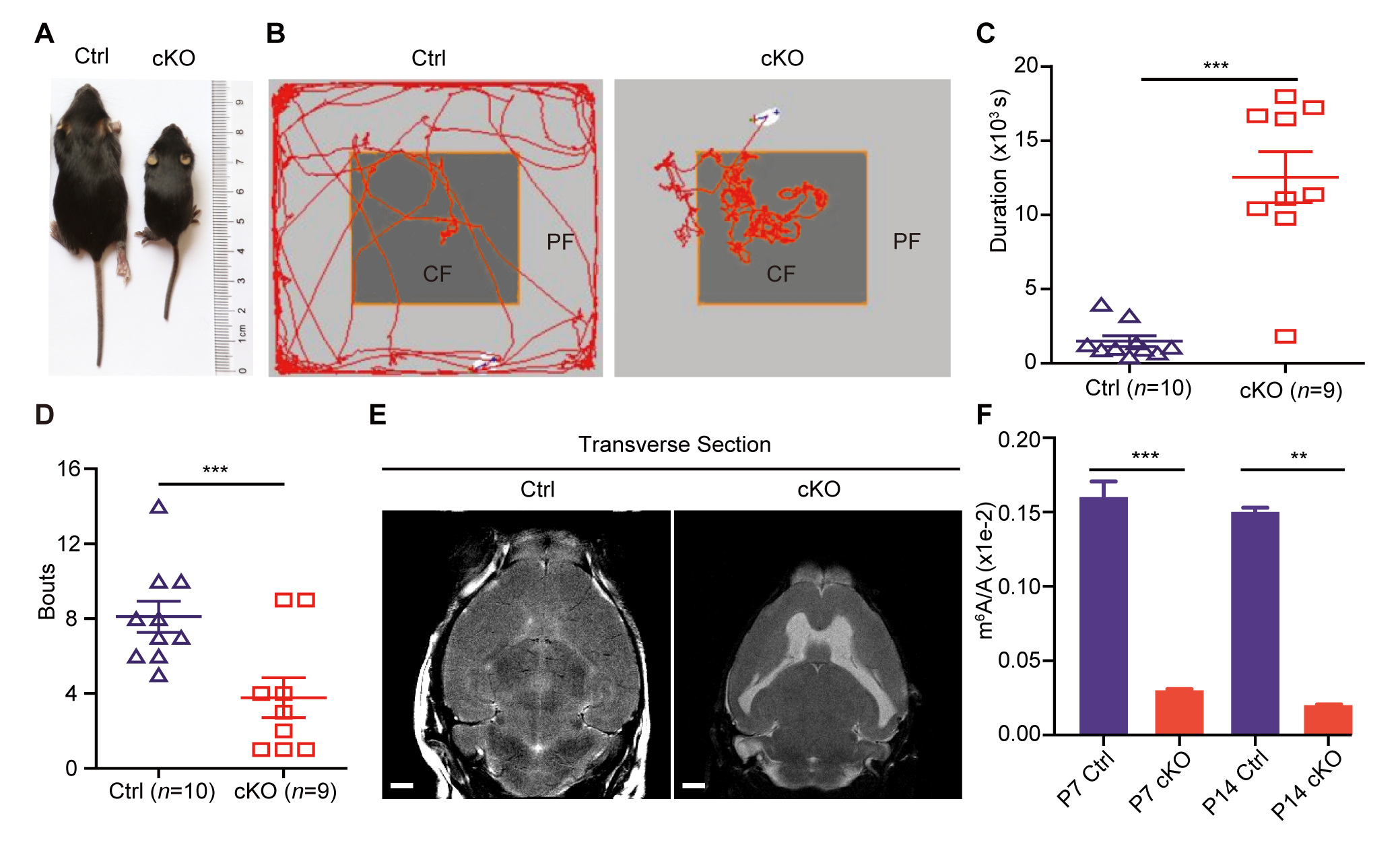

Supplement: S2 Fig — (A) Body size comparison of the Ctrl and cKO mice at P14. (B–D) Open field test shows the representative activity traces (B), time spent in the central region (C), and bouts from the central field to the peripheral field (D) for both Ctrl and cKO mice at P14. (E) MRI analysis for the whole brain transverse section of the Ctrl and cKO mice at P14. Scale bar, 1 mm. (F) UHPLC-MS/MS quantification of m6A levels in mRNAs isolated from the Ctrl and cKO mouse brain at P7 and P14. Further information about this figure can be found in S1 Data. The data were represented as means ± SEM. Three technical measurements from two biological replicates were performed. **p-value < 0.01, ***p-value < 0.001, Student t test. CF, central field; cKO, Mettl3 conditional knockout; Ctrl, control; Mettl3, methyltransferase-like 3; MRI, magnetic resonance imaging; PF, peripheral field; SEM, standard error of the mean; UHPLC-MS/MS, ultra high-pressure liquid chromatography tandem-mass spectrometry. (TIF) [file pbio.2004880.s002.tif]

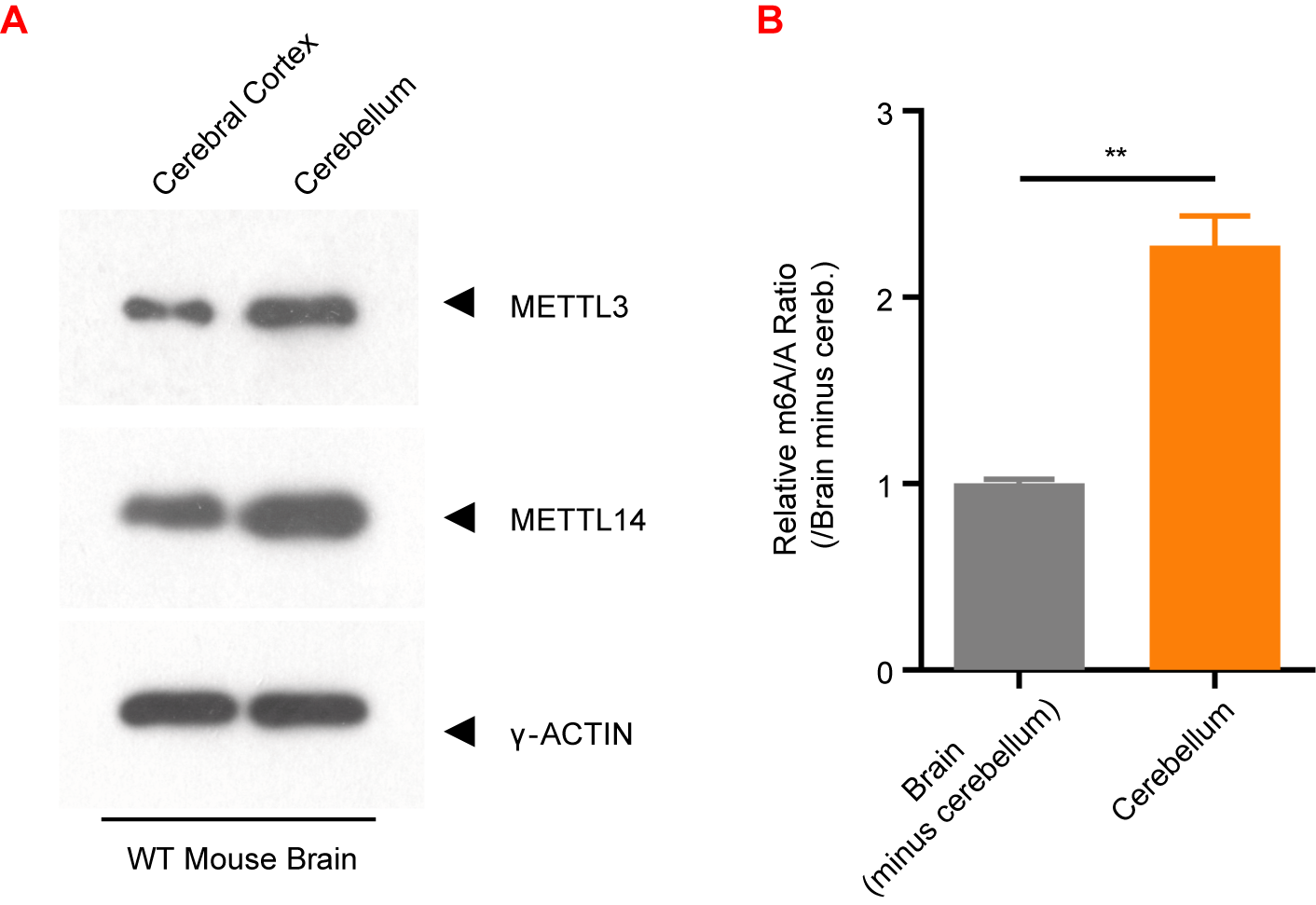

Supplement: S3 Fig — (A) Western blot comparing the expression of METTL3 and METTL14 between the cerebellum and cerebral cortex of wild-type mice. (B) UHPLC-MS/MS quantification of m6A levels in mRNAs isolated from the cerebellum and brain regions minus the cerebellum. Data related to this figure are shown in S1 Data. Data shown are means ± SEM. Three technical measurements from two biological replicates were performed. **p-value < 0.01, Student t test. γ-ACTIN, loading control; METTL3, methyltransferase-like 3; METTL14, methyltransferase-like 14; m6A, N6-methyladenosine; SEM, standard error of the mean; UHPLC-MS/MS, ultra high-pressure liquid chromatography tandem-mass spectrometry. (TIF) [file pbio.2004880.s003.tif]

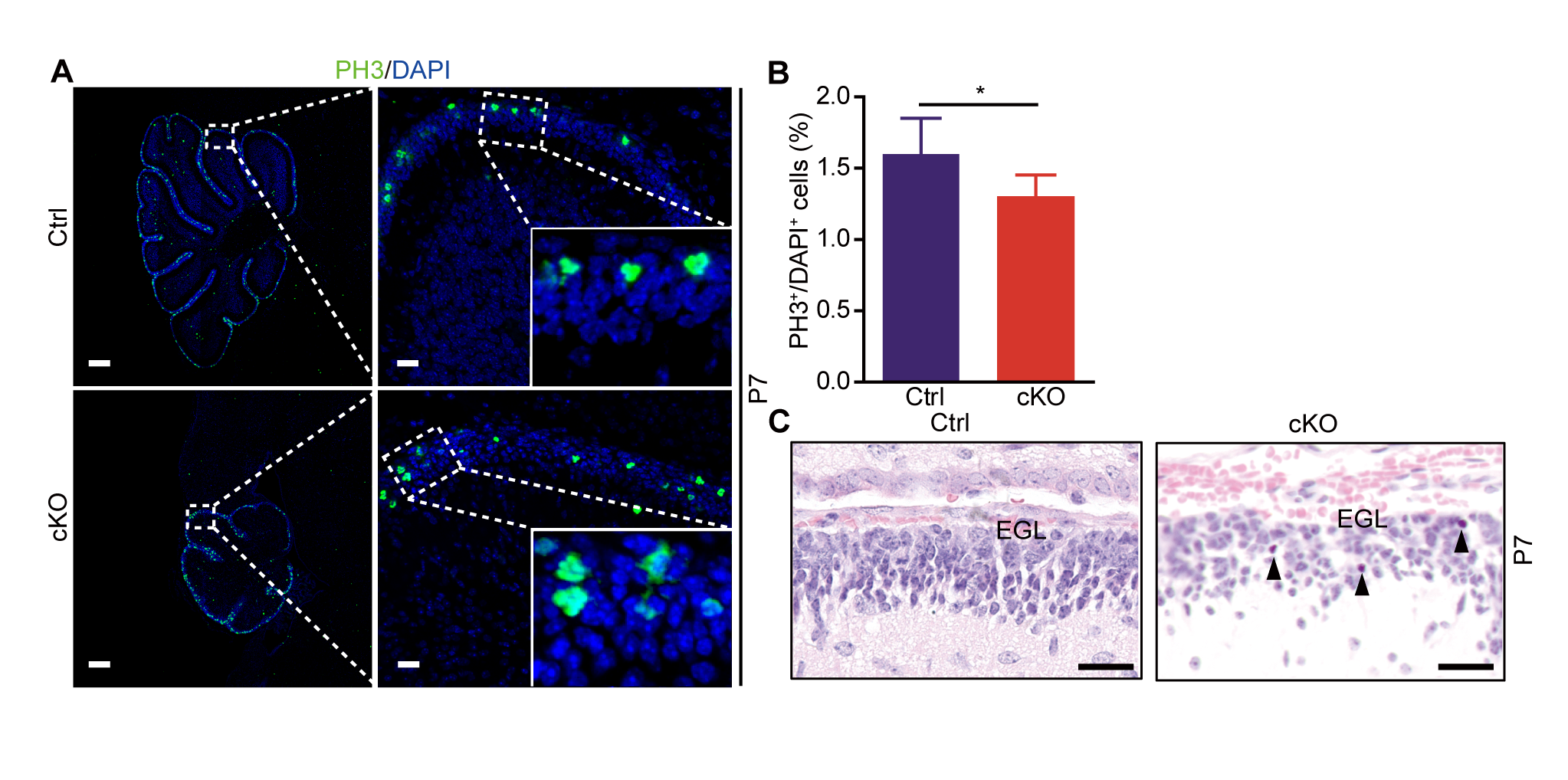

Supplement: S4 Fig — (A–C) Immunohistochemical analysis of Mettl3 in the cerebellum at E16.5, P7, and P14. Scale bar, 200 μm. (D) HE staining and immunohistochemical analysis of Ctrl and cKO cerebellums at P7. Scale bar, 200 μm. cKO, Mettl3 conditional knockout; Ctrl, control; EGL, external granular layer; HE, hematoxylin and eosin; IGL, internal granular layer; ML, molecular layer; PCL, Purkinje cell layer. (TIF) [file pbio.2004880.s004.tif]

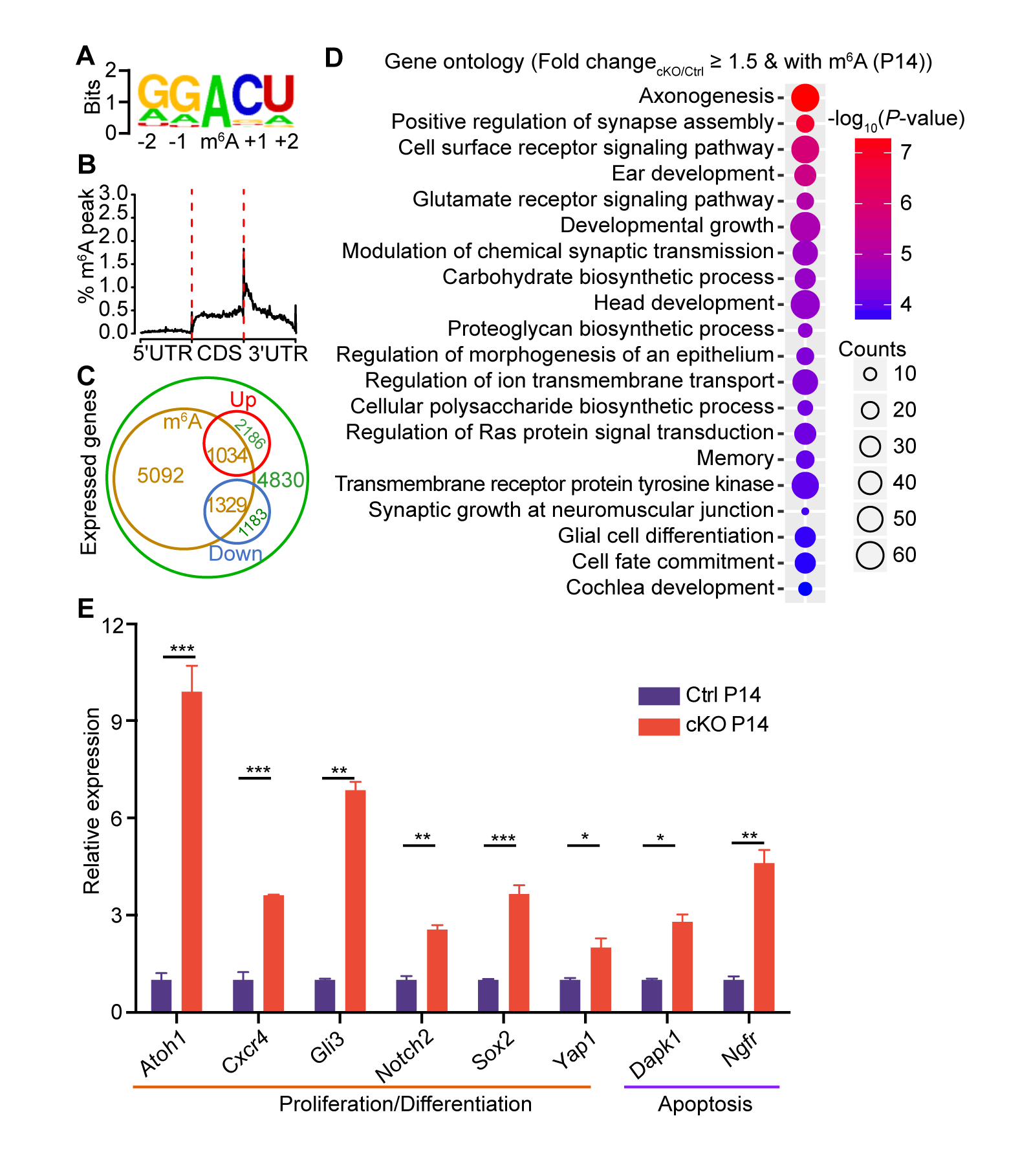

Supplement: S5 Fig — (A) Immunofluorescent staining of PH3 (green) and DAPI (blue) in cerebellums of Ctrl and cKO mice at P7. Scale bar for the left panels, 200 μm. Scale bar for the right panels, 25 μm. (B) Proportion of PH3+/DAPI+ cells in the EGL of Ctrl and cKO mice at P7. Further information about this figure can be found in S1 Data. The data were represented as means ± SEM; n = 3 for each group. *p-value < 0.05, Student t test. (C) High magnificent HE staining images of EGL cells from Ctrl and cKO mice at P7. Black triangles indicate cells with karyorrhexis and karyopyknosis. Scale bar, 200 μm. cKO, Mettl3 conditional knockout; Ctrl, control; EGL, external granular layer; HE, hematoxylin and eosin; PH3, phosphorylated H3; SEM, standard error of the mean. (TIF) [file pbio.2004880.s005.tif]

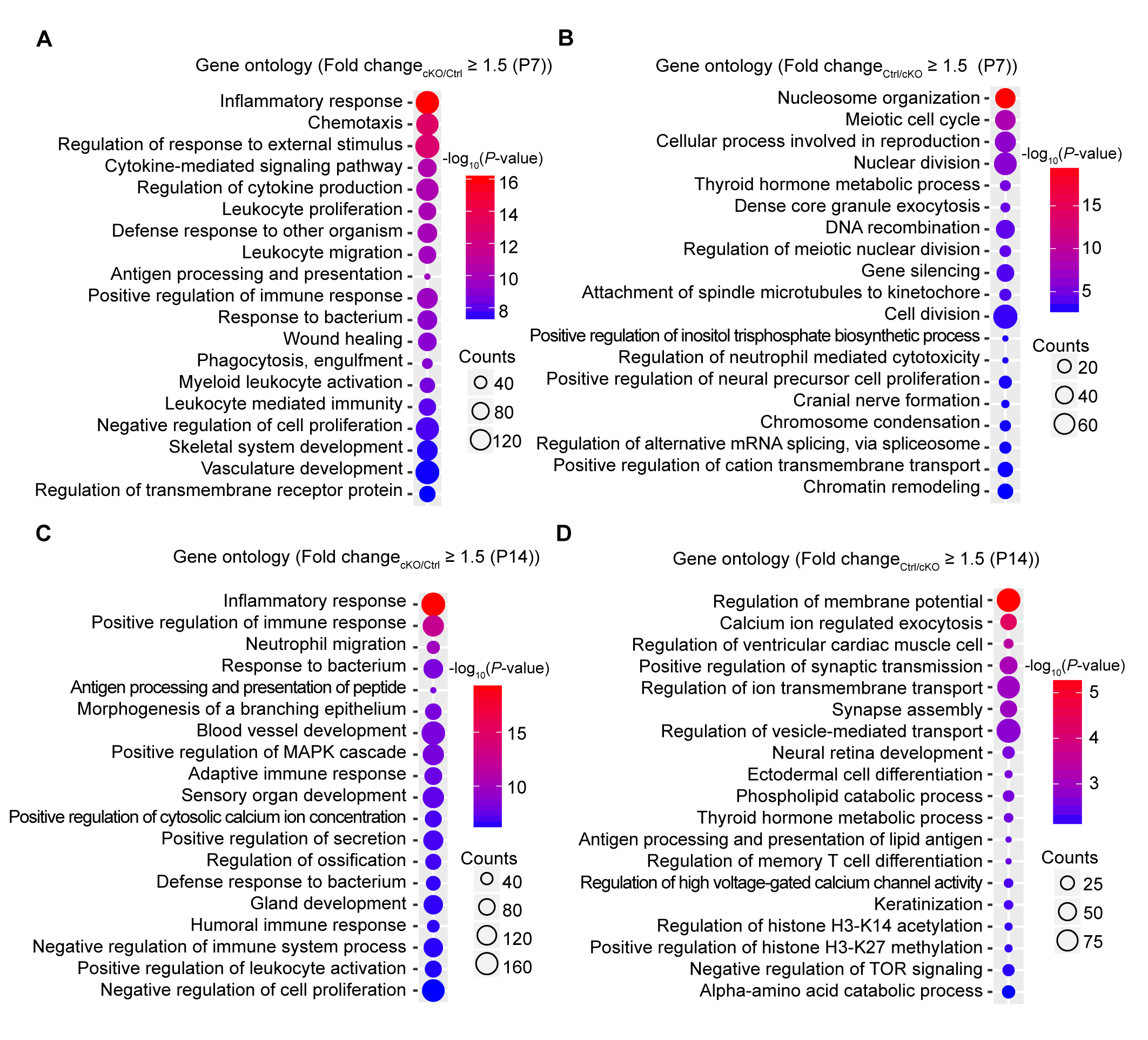

Supplement: S6 Fig — (A) The most enriched sequence motif of m6A peaks in RNAs from P14 mouse cerebellum. (B) Distribution of m6A peaks along the 5′UTR, CDS, and 3′UTR regions of total cerebellar mRNAs from P14 Ctrl mice after normalized with length. (C) Venn diagram representing the relationships between cerebellar expressed genes and m6A modification. Green circle represents all expressed genes in P14 Ctrl mouse cerebellum, brown circle represents genes with m6A modifications in P14 Ctrl mouse cerebellum, red circle represents genes with up-regulated expression in P14 Mettl3 cKO mouse cerebellum as compared to the Ctrl, and blue circle represents genes with down-regulated expression in P14 Mettl3 cKO mouse cerebellum as compared to the Ctrl. Numbers represent the counts of genes in each group. (D) Significantly enriched (p-value ≤ 0.01, Banjamini–Hochberg multiple testing correction) GO terms of genes with up-regulated expression in P14 cKO cerebellums and with m6A peaks. (E) qRT-PCR results confirmed the up-regulated expression in P14 cKO cerebellums of selected genes. The major functions of detected genes are shown under the lines. Further information about this figure can be found in S1 Data. The data were represented as means ± SEM; n = 3. *p-value < 0.05, **p-value < 0.01, ***p-value < 0.001, Student t test. CDS, coding sequence; cKO, Mettl3 conditional knockout; GO, gene ontology Mettl3, methyltransferase-like 3; m6A, N6-methyladenosine; qRT-PCR, quantitative reverse transcription-polymerase chain reaction; SEM, standard error of the mean. (TIF) [file pbio.2004880.s006.tif]

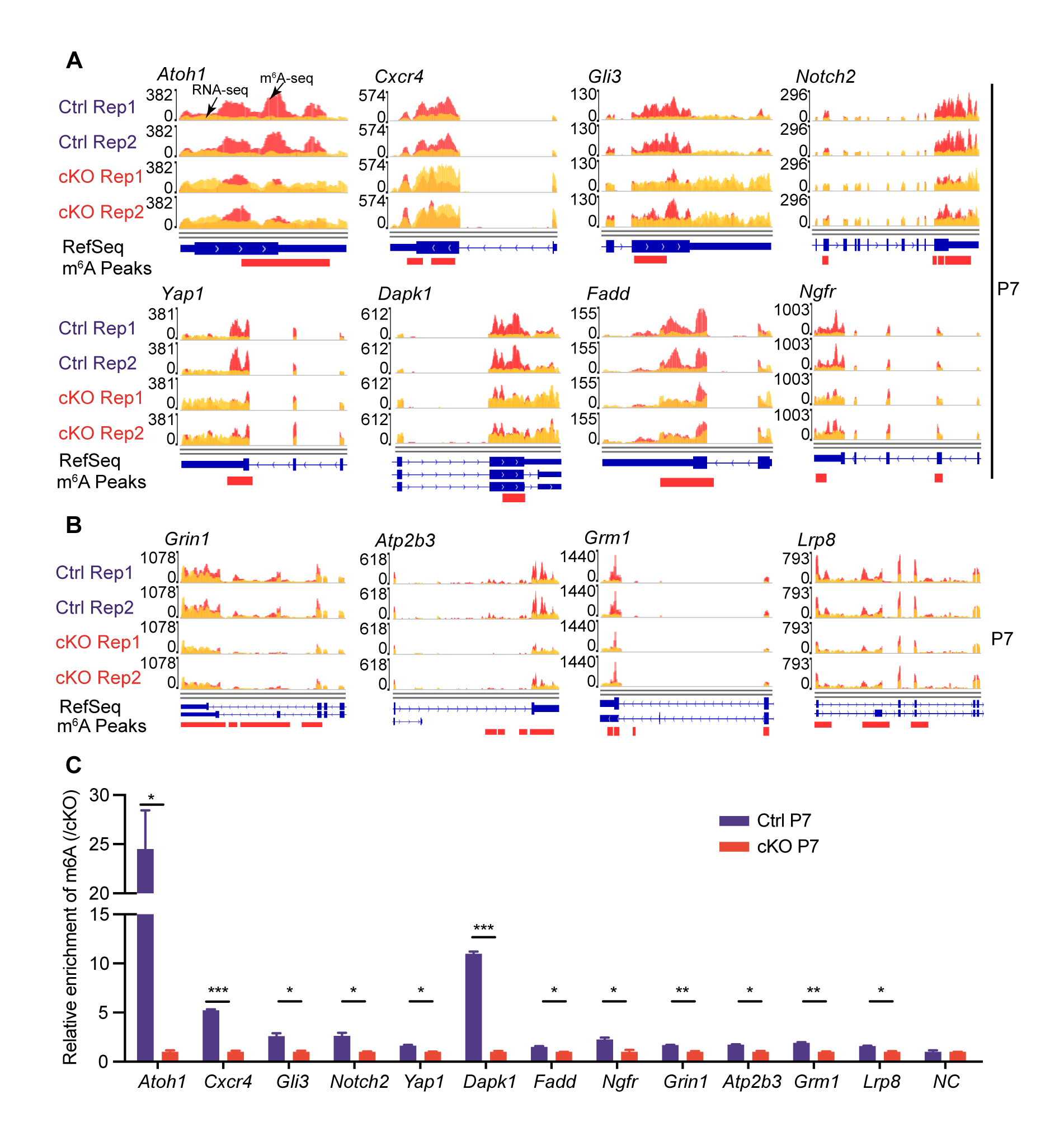

Supplement: S7 Fig — (A–D) Enriched GO terms (p-value ≤ 0.01, Benjamini–Hochberg multiple testing correction) of differentially up-regulated genes in P7 cKO (A), down-regulated genes in P7 cKO (B), up-regulated genes in P14 cKO (C), and down-regulated genes in P14 cKO (D) as compared with the paired Ctrl. cKO, Mettl3 conditional knockout; GO, gene ontology. (TIF) [file pbio.2004880.s007.tif]

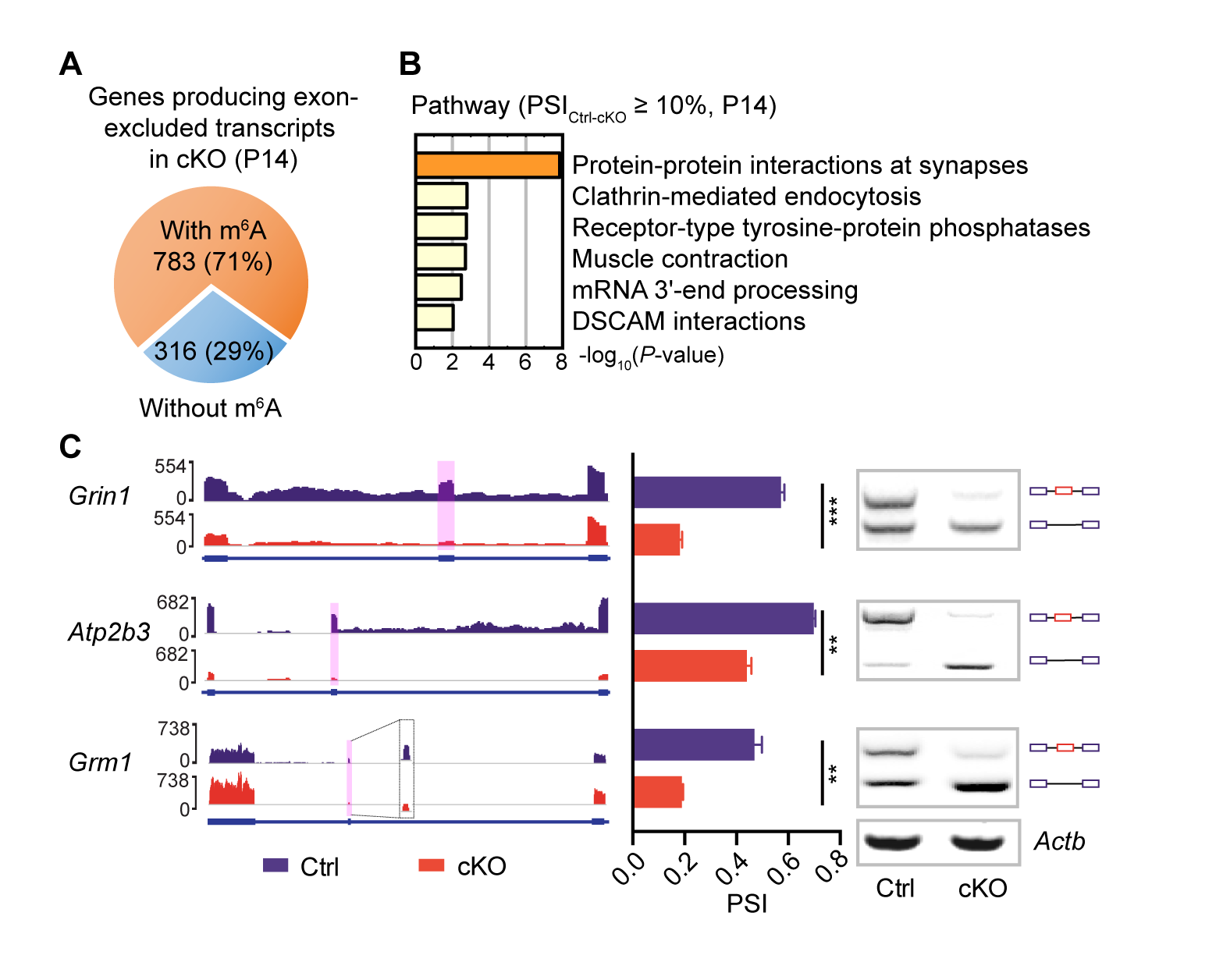

Supplement: S8 Fig — (A–B) The m6A peak plots of the differentially expressed genes (A) and the alternatively spliced genes (B) in Mettl3 cKO mice. The abundance of normalized m6A-seq data and normalized RNA-seq data are shown in red and yellow, respectively. (C) MeRIP qRT-PCR results confirmed the depletion of m6A modification in the differentially expressed genes and alternatively spliced genes in the cKO cerebellums. Further information about this figure can be found in S1 Data. The data were represented as means ± SEM. *p-value < 0.05, **p-value < 0.01, ***p-value < 0.001. cKO, Mettl3 conditional knockout; m6A, N6-methyladenosine; m6A-seq, m6A sequencing; MeRIP, methylated RNA immunoprecipitation; Mettl3, methyltransferase-like 3; qRT-PCR, quantitative reverse transcription polymerase chain reaction; RNA-seq, RNA-sequencing; SEM, standard error of the mean. (TIF) [file pbio.2004880.s008.tif]

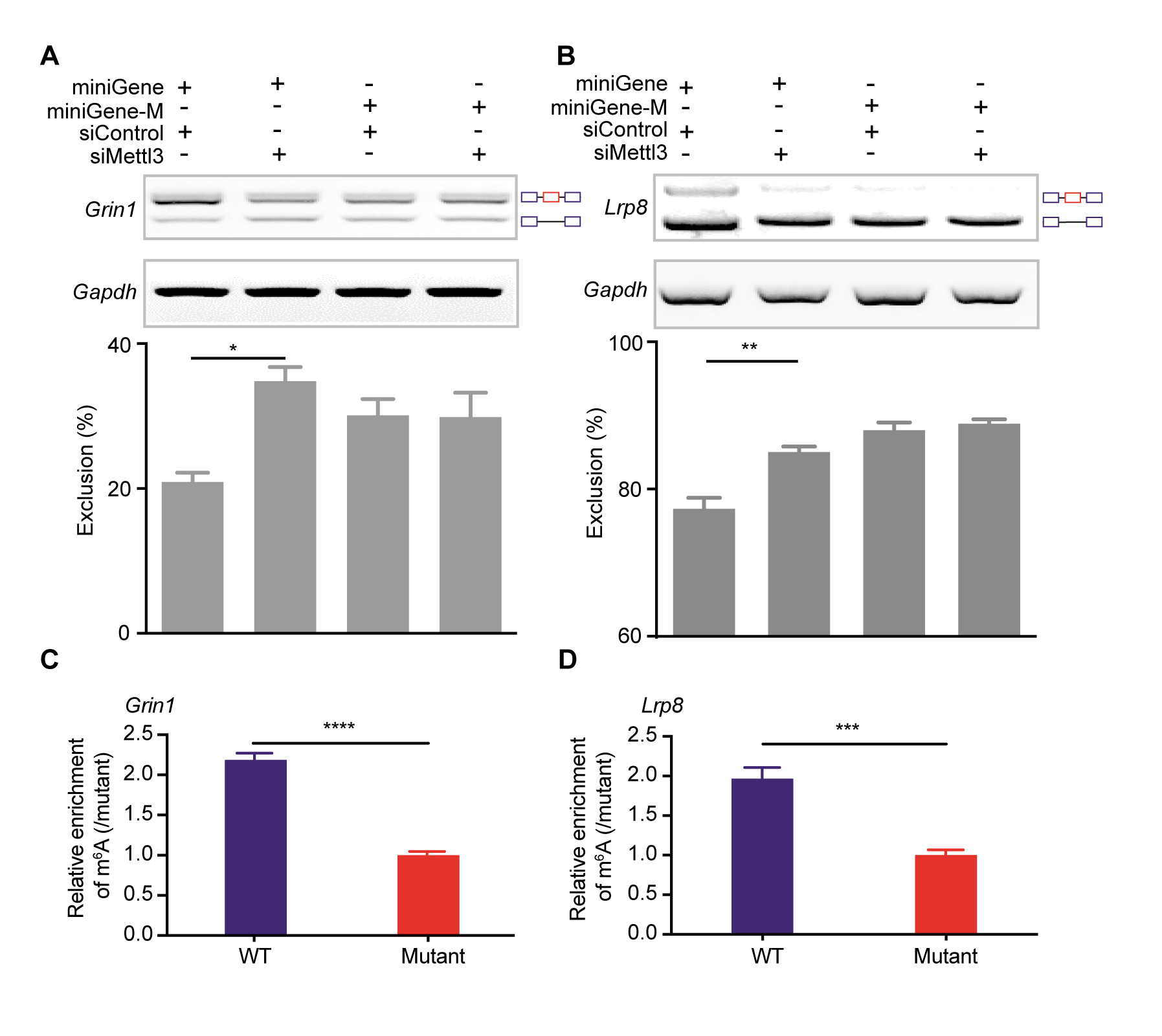

Supplement: S9 Fig — (A) Genes producing exon-excluded transcripts in P14 cKO cerebellums as compared with the Ctrl. (B) Enriched pathways of genes producing exon-excluded transcripts in P14 cKO cerebellums as compared with the Ctrl. (C) Production of exon-excluded transcripts of selected genes in P14 cKO cerebellums. Left, IGV tracks displaying the RNA-seq reads coverage in P7 Ctrl (blue) and cKO (red) cerebellum; the excluded exons are shaded with pink columns. Y-axis: normalized reads counts. Middle, calculated exon-exclusion percentage in P7 cKO cerebellums by the PSI value. Right, semiquantitative PCR detection of exon-excluded transcripts in P7 cKO cerebellums. Further information about this figure can be found in S1 Data. The data were represented as means ± SEM. **p-value < 0.01, ***p-value < 0.001, Student t test. cKO, Mettl3 conditional knockout; Ctrl, control; IGV, integrative genomics viewer; Mettl3, methyltransferase-like 3; m6A, N6-methyladenosine; PSI, percent spliced in index; RNA-seq, RNA-sequencing; SEM, standard error of the mean. (TIF) [file pbio.2004880.s009.tif]

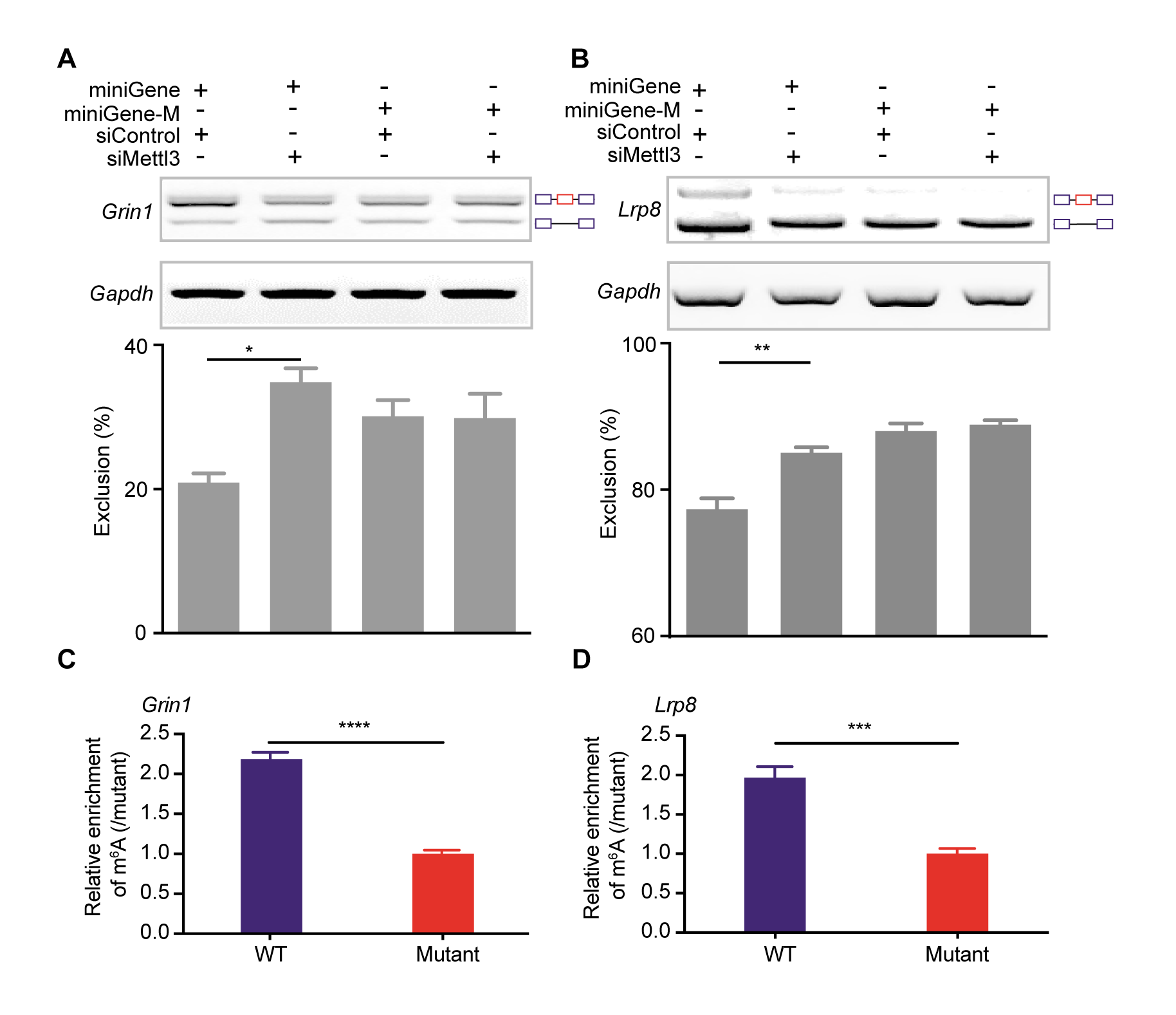

Supplement: S10 Fig — (A–B) The exclusion level of exon 21 of Grin1 (A) and exon 19 in Lrp8 (B) was validated by RT-PCR in HeLa cells transfected with (1) siControl and miniGene, (2) siMettl3 and miniGene, (3) siControl and miniGene-M, and (4) siMettl3 and miniGene-M. (C–D) MeRIP qRT-PCR results confirmed the depletion of m6A modification on the alternatively spliced exon of Grin1(C) and Lrp8 (D), with mutations in the predicted m6A modification sites. Further information about this figure can be found in S1 Data. Values and error bars in all plots represent the mean and SEM of three independent experiments by Student t test. *p-value < 0.05, **p-value < 0.01, ***p-value < 0.001, ****p-value < 0.0001. Grin1, glutamate ionotropic receptor NMDA type subunit 1; Lrp8, low density lipoprotein receptor-related protein 8; MeRIP, methylated RNA immunoprecipitation; miniGene-M, miniGene-Mutant; m6A, N6-methyladenosine; qRT-PCR, quantitative reverse transcription polymerase chain reaction; SEM, standard error of the mean; siControl, control siRNA; siMettl3, Mettl3 siRNA. (TIF) [file pbio.2004880.s010.tif]
